# Evolution of Chloroplast Transcript Processing in *Plasmodium* and Its Chromerid Algal Relatives

**DOI:** 10.1371/journal.pgen.1004008

**Published:** 2014-01-16

**Authors:** Richard G. Dorrell, James Drew, R. Ellen R. Nisbet, Christopher J. Howe

**Affiliations:** 1 Department of Biochemistry, University of Cambridge, Cambridge, United Kingdom; 2 School of Pharmacy and Medical Sciences, University of South Australia, Adelaide, Australia; Washington University School of Medicine, United States of America

## Abstract

It is well understood that apicomplexan parasites, such as the malaria pathogen *Plasmodium*, are descended from free-living algae, and maintain a vestigial chloroplast that has secondarily lost all genes of photosynthetic function. Recently, two fully photosynthetic relatives of parasitic apicomplexans have been identified, the ‘chromerid’ algae *Chromera velia* and *Vitrella brassicaformis*, which retain photosynthesis genes within their chloroplasts. Elucidating the processes governing gene expression in chromerid chloroplasts might provide valuable insights into the origins of parasitism in the apicomplexans. We have characterised chloroplast transcript processing pathways in *C. velia*, *V. brassicaformis* and *P. falciparum* with a focus on the addition of an unusual, 3′ poly(U) tail. We demonstrate that poly(U) tails in chromerids are preferentially added to transcripts that encode proteins that are directly involved in photosynthetic electron transfer, over transcripts for proteins that are not involved in photosynthesis. To our knowledge, this represents the first chloroplast transcript processing pathway to be associated with a particular functional category of genes. In contrast, *Plasmodium* chloroplast transcripts are not polyuridylylated. We additionally present evidence that poly(U) tail addition in chromerids is involved in the alternative processing of polycistronic precursors covering multiple photosynthesis genes, and appears to be associated with high levels of transcript abundance. We propose that changes to the chloroplast transcript processing machinery were an important step in the loss of photosynthesis in ancestors of parasitic apicomplexans.

## Author Summary

Chloroplasts contain their own genomes, containing two broad functional types of gene: genes encoding proteins directly involved in photosynthesis, and genes with a non-photosynthesis function, such as cofactor biosynthesis, assembly of protein complexes, or expression of the chloroplast genome. Thus far, to our knowledge, no chloroplast gene expression pathways in any lineage have been found to target one functional category of gene specifically. Here, we show that a chloroplast RNA processing pathway – the addition of a 3′ poly(U) tail – is specifically associated with photosynthesis genes in two species of algae, the ‘chromerids’ *Chromera* and *Vitrella*. The addition of the poly(U) tail enables the precise processing of mature photosynthesis gene transcripts from precursor RNA, and is likely to be essential for expression of the chromerid photosynthesis machinery. The chromerid algae are the closest photosynthetic relatives of a parasitic group of eukaryotes, the apicomplexans, which include the malaria pathogen *Plasmodium*. Apicomplexans are descended from algae, and retain a reduced chloroplast, which contains genes only of non-photosynthesis function. We have confirmed that 3′ poly(U) tails are not added to *Plasmodium* chloroplast transcripts. The expression pathways associated with photosynthesis genes have therefore been lost in the evolution of the apicomplexan chloroplast, and this loss could potentially have driven the transition from photosynthesis to parasitism.

## Introduction

The transition from a photosynthetic to a parasitic lifestyle has occurred a multitude of times across the eukaryotes [1]. Parasitism, concomitant with either the complete loss or a severe reduction in dependence on photosynthesis, has been documented in members of the land plants and the green, red, and brown algae [1]–[4]. Typically, parasitic organisms descended from photosynthetic ancestors retain chloroplasts with their own genome, but these genomes are vastly reduced in content. Various hypotheses have been suggested for why certain genes are retained in the chloroplast, and others are transferred to the nucleus, such as the greater relative frequency of mutations in chloroplast genes, the higher energetic cost associated with synthesis and import of cytoplasmic proteins, and the direct regulation of chloroplast-encoded genes in response to changes in chloroplast redox state, or other biochemical parameters [5]–[7]. However, the reasons why, and when, chloroplast genes are lost during the transition from photosynthesis to parasitism remain comparatively underexplored.

Perhaps the most dramatic example of the transition from photosynthesis to parasitism occurs in the apicomplexans, a group containing several pathogens of major humanitarian importance, including the malaria parasite *Plasmodium*, and *Toxoplasma* and *Cryptosporidium*, causative agents respectively of toxoplasmosis and cryptosporidiosis, both potentially fatal to immuno-compromised patients [1]. Apicomplexans are descended from photosynthetic algae, and the majority - apart from *Cryptosporidium* - retain a vestigial, non-photosynthetic, chloroplast-derived organelle, termed the ‘apicoplast’, which is involved in a number of metabolic pathways fundamental to parasite viability and pathology [8], [9]. The apicoplast contains its own genome that is conventionally organised, but has lost all genes for proteins that function directly in photosynthetic electron transfer, which we will henceforth term ‘photosynthesis genes’, and only retains genes of non-photosynthetic function [10].

Although the evolutionary origin of the apicoplast has been the subject of debate, recent studies firmly place it as being of secondary, red algal derivation, and sharing a common ancestry with the chloroplasts found in a closely related group of algae, the peridinin dinoflagellates [10]–[12]. Peridinin dinoflagellates possess an extremely unusual chloroplast genome, which is organised on small, plasmid-like elements termed ‘minicircles’, and has a highly reduced coding content. The chloroplast genomes of peridinin dinoflagellates contain only a few photosynthesis genes, genes for ribosomal and transfer RNAs, and in some species other open reading frames that lack an identified function, or recognisable homologues to other lineages [1], [13]. Dinoflagellate chloroplasts use abnormal RNA metabolism pathways including rolling circle transcription, and extensive post-transcriptional editing occurs in certain species [14], [15]. Most dramatically, a 3′ terminal poly(U) tail is added during the processing of transcripts of protein-coding genes [16]. While similar transcript polyuridylylation events have been reported in various nuclear and mitochondrial lineages, they do not occur in plant or other algal chloroplasts. Transcript polyuridylylation has likewise not been found in the apicoplast, although, to our knowledge, this has not been shown systematically and the only information available comes indirectly from EST libraries and next generation sequencing reads [12], [17], [18].

In the past decade, two fully photosynthetic close relatives of apicomplexan parasites have been identified, jointly referred to as the ‘chromerid’ algae [1], [19]. *Chromera velia* is a small, single-celled alga with coccoid and motile forms, whereas *Vitrella brassicaformis* (e.g. CCMP3155) is a much larger, pseudocolonial alga, with a complex life cycle [19], [20]. Both species form symbiotic associations with zooxanthellate corals [19]–[21], and recent studies suggest that many more as yet uncultured chromerids are present in coralline environments [22], [23]. Phylogenetic analyses robustly place *C. velia* and *V. brassicaformis* as separate sister groups to apicomplexans [11], [19] to the exclusion of peridinin dinoflagellates. The exact dates at which the chromerid lineages diverged from the parasitic apicomplexans remain a matter of debate, although these have been estimated to be anywhere between 350 and 700 million years before the present, and were probably well after the divergence of the common ancestor of apicomplexans and chromerids from the dinoflagellates [24]–[26]. Some of the metabolic pathways associated with the chloroplasts of *C. velia*, at least, are more similar to those occurring in the chloroplasts of other free-living algae than to those of apicomplexans, whereas the opposite is true for others [27]–[31]. A more detailed understanding of the processes governing the expression of photosynthesis versus non-photosynthesis genes in the chloroplasts of chromerid algae might provide insights into the evolution of parasitism in early apicomplexans.

The chloroplast genomes of both *C. velia* and *V. brassicaformis* have been sequenced and are of the same endosymbiotic derivation as those of apicomplexans and peridinin dinoflagellates. The *V. brassicaformis* chloroplast genome consists of one single circular chromosome, similar to that of apicomplexans, while the *C. velia* chloroplast genome is believed to comprise a single, long linear chromosome, unlike that of either dinoflagellates or apicomplexans [11], [32]. However, both chromerid chloroplast genomes are larger than those of either the apicomplexan or peridinin dinoflagellate lineages, retaining 55 (*Chromera*) and 71 (*Vitrella*) non-redundant annotated protein-coding genes, of both photosynthetic and non-photosynthetic function, as well as a small number of open reading frames of unannotated function, and specific to either species [11].

It has previously been shown that, similar to the situation in dinoflagellates, poly(U) tails are added to at least three chloroplast transcripts (*psaA*, *psbB*, *psbC*) in *C. velia* [11], [32]. However, it is not known to which other transcripts in *C. velia* chloroplasts poly(U) tails are added, or whether similar poly(U) addition occurs in *V. brassicaformis* [32]. More broadly, the precise functional role of transcript poly(U) addition in chromerid algae remains uncharacterised. Here, we present an in-depth study of transcript poly(U) addition in *C. velia* and *V. brassicaformis*. We demonstrate that in both species poly(U) tails are principally added to transcripts encoding functional components of the photosynthetic electron transfer chain. Conversely, transcripts that do not encode products that directly function in photosynthesis tend not to be polyuridylylated in either *C. velia* or *V. brassicaformis*. This is to our knowledge the first example of a chloroplast transcript processing pathway that differentially recognises a particular functional category of genes. We additionally demonstrate that poly(U) addition occurs early in transcript processing in *C. velia*, and may influence other processing events on photosynthesis gene transcripts. Finally, we confirm that poly(U) addition does not occur in the apicomplexan *Plasmodium falciparum.* As the poly(U) machinery in chromerid algae is involved in the differential recognition of photosynthesis and non-photosynthesis genes, its loss may have played an important role in the transition of early apicomplexans from photosynthesis to obligate parasitism.

## Results

### Poly(U) tails are principally added to photosynthesis gene transcripts in *Chromera velia* and *Vitrella brassicaformis*

**Figure 1:**
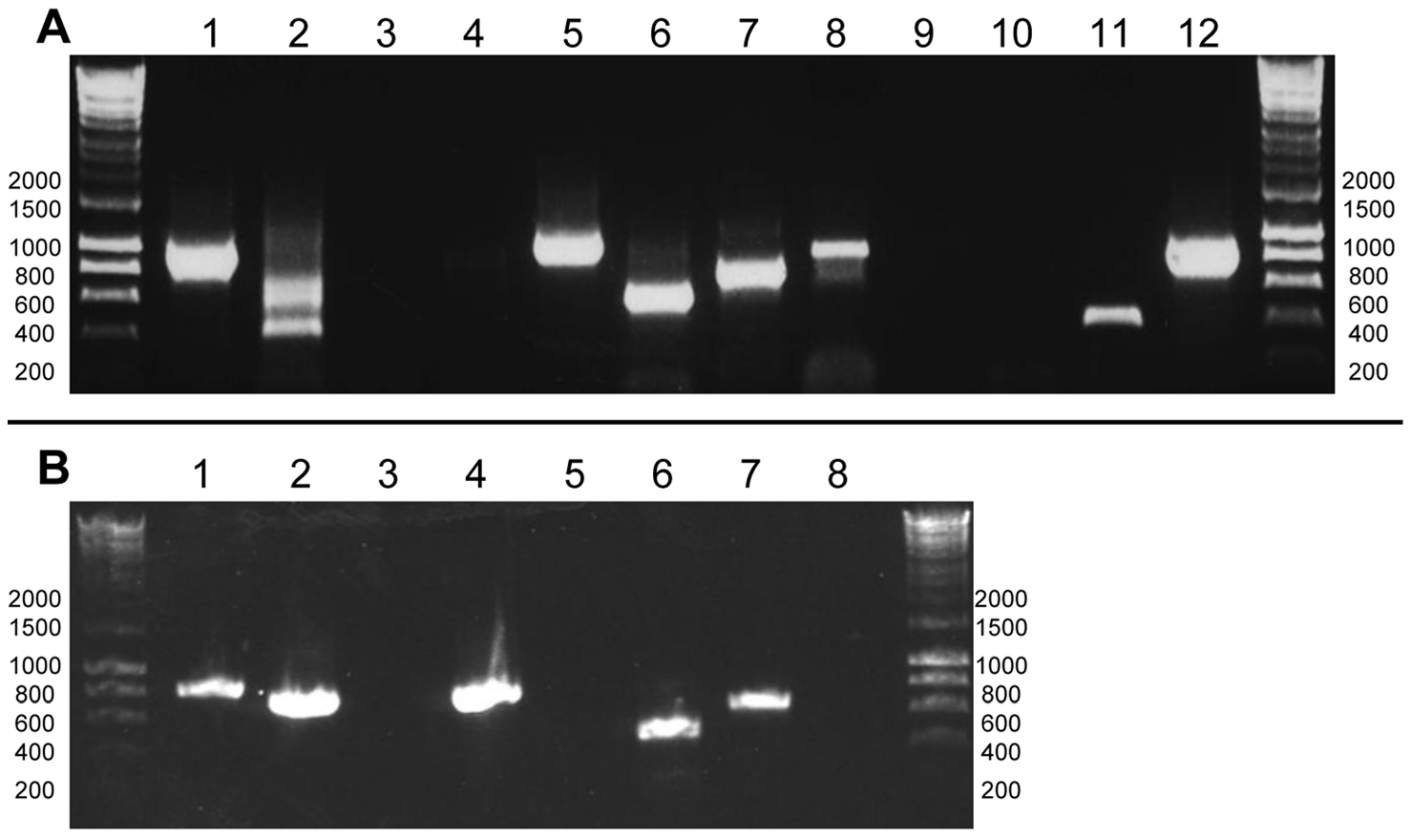
Determining the poly(U) state of representative chloroplast genes from *C. velia* and *V. brassicaformis*. Hyperladder I (Bioline) was used as a size marker, with the positions of representative size bands given to the side of each gel photo. Panel A: RT-PCRs for *Chromera velia* (lanes 1–6) and *Vitrella brassicaformis* (lanes 7–12). Lanes 1–2, 7–8: Oligo-d(A) RT-PCRs for the photosynthesis genes *psbA* and *atpB-2* (*C. velia)/atpB* (*V. brassicaformis*); Lanes 3–4, 9–10: oligo-d(A) RT-PCRs for the non-photosynthesis genes *rps11* and *rrs*; Lanes 5–6, 11–12: RT-PCRs using an internal, gene-specific cDNA primer for *rps11* and *rrs* for both species. The multiple bands observed for *C. velia atpB-2* (lane 2) correspond to different *atpB(2)* transcripts containing alternative poly(U) sites. Panel B control RT-PCRs. Lanes 1–2: oligo-d(A) and internal gene-specific RT-PCRs for *Amphidinium carterae p*s*bA*; lanes 3–4: oligo-d(A) and internal gene-specific RT-PCRs for *Phaeodactylum tricornutum psbA*; Lanes 5–6: oligo-d(A) and internal gene-specific RT-PCRs for *C. velia Hsp90*; lanes 7–8: PCR positive (DNA template) and negative controls (no template) for *C. velia psbA*.

To test for the presence of polyuridylylated transcripts in *C. velia* and *V. brassicaformis*, we generated cDNA using an oligo-d(A) primer, which anneals to transcript poly(U) tails [12], [33]. The oligo-d(A) primed cDNA was used as a template for a series of PCR reactions using the same oligo-d(A) primer, and a series of forward primers specific to different chloroplast genes from each species (Table S1). Figure 1 shows the results from two representative photosynthesis genes where RT-PCR products were obtained, consistent with the presence of polyuridylylated transcripts (fig. 1: *C. velia psbA*, *atpB-2*, panel A, lanes 1–2; *V. brassicaformis psbA*, *atpB*, panel A, lanes 7–8). The identity of each transcript obtained via PCR was confirmed by direct sequencing, using the PCR forward primer as the sequencing primer. Similar products were observed with a control transcript from a dinoflagellate chloroplast (*Amphidinium carterae psbA*) that is known to be polyuridylylated (fig. 1, panel B, lane 1) [33].

In contrast, analogous RT-PCRs against representative chloroplast genes that do not encode products directly involved in photosynthesis from both species (*rps11* and *rrs*) failed to resolve clear products, even after two successive rounds of PCR amplification (fig. 1, panel A, lanes 3–4, 9–10). We could detect RT-PCR products generated using gene-specific primers (fig. 1, panel A lanes 5–6, 11–12, Table S2), implying that transcripts of each gene are present, but do not receive a poly(U) tail. Similar products were observed for a control nuclear transcript (*C. velia Hsp90*) as well as a transcript from a diatom chloroplast (*Phaeodactylum tricornutum psbA*) which has previously been shown not to receive a poly(U) tail (fig. 1, panel B lanes 3–6) [12].

**Figure 2:**
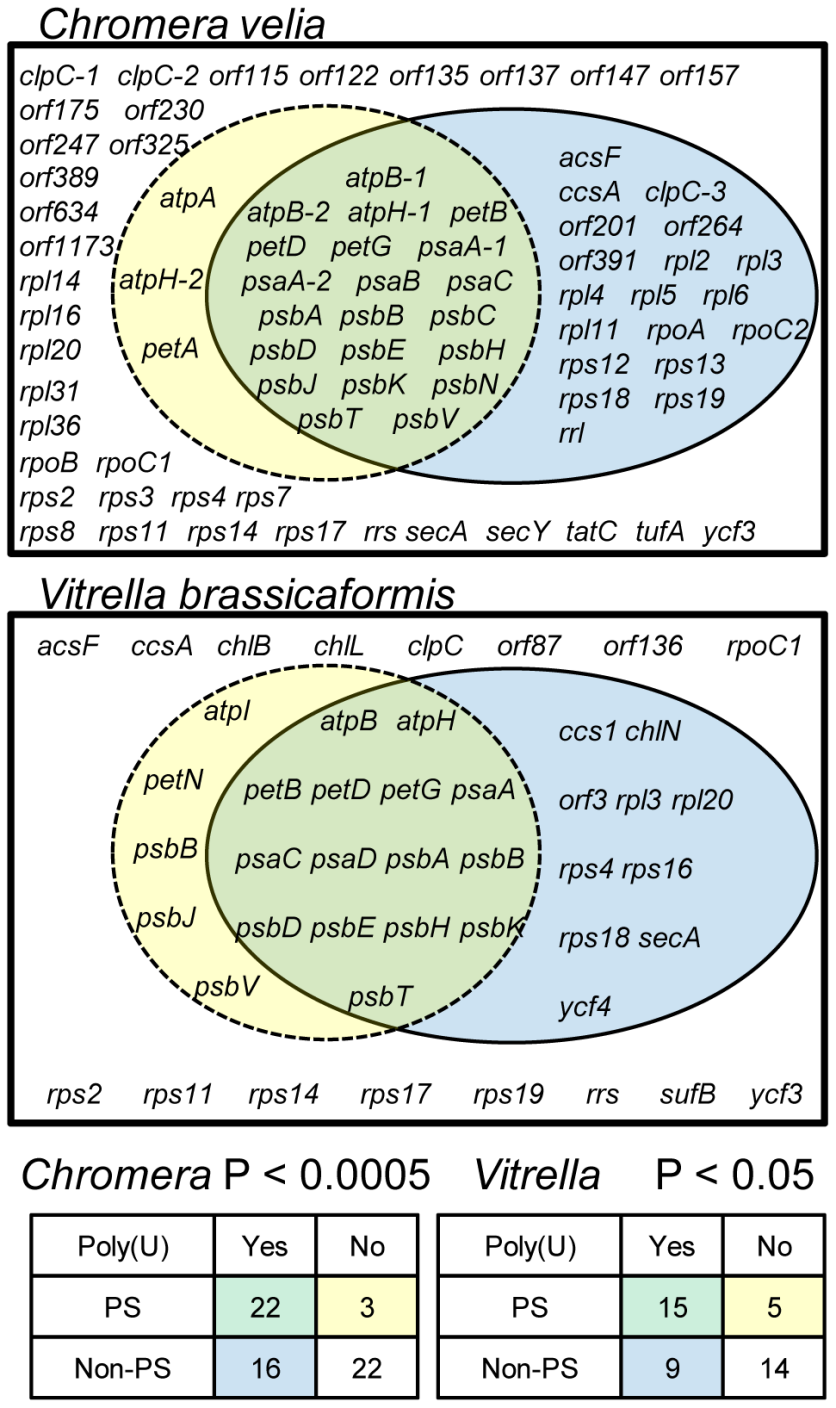
The total distribution of poly(U) sites across chromerid chloroplasts. The Venn diagrams show the total results of oligo-d(A) RT-PCRs for genes from *C. velia* and *V. brassicaformis*. Chi-squared distributions and P values for the significance of association between photosynthesis function and presence of an associated poly(U) site are shown at the bottom of the diagram.

To determine whether poly(U) tail addition is significantly biased towards photosynthesis genes in chromerid algae, we performed oligo-d(A) RT-PCRs against every annotated gene and open reading frame in the *C. velia* chloroplast genome (n = 78), and over half the genes in the *V. brassicaformis* chloroplast genome (n = 43, out of 74 total) (Table S2). In both species, transcript poly(U) addition was significantly biased towards photosynthesis genes (chi-squared: *C. velia* P<0.005; *V. brassicaformis* P<0.05). While we could identify some genes that contradicted general patterns - i.e. transcripts of photosynthesis genes that do not receive a poly(U) tail or polyuridylylated transcripts that encode non-photosynthesis proteins, or novel open reading frames – most of these exceptions were specific to one species. Only two non-photosynthesis genes (*rpl3* and *rps18*) were found to possess poly(U) sites in both species, and none of the non-polyuridylylated photosynthesis genes was conserved between *C. velia* and *V. brassicaformis* (fig. 2). None of the poly(U) sites identified was predicted to lie within poly(T) tracts of more than 6 bp in either genomic sequence, suggesting that the poly(U) tails are not genome-encoded [11]. Consistent with other studies [32], we could not detect any evidence of post-transcriptional editing in any transcripts in either species.

### Location of poly(U) sites

We wished to determine whether poly(U) sites were associated with specific regions of chromerid chloroplast genomes. Comparison of RT-PCR products with genomic sequences showed that, other than a preferential association with photosynthesis gene 3′ UTRs, poly(U) sites are broadly distributed across chromerid chloroplast genomes. We could identify poly(U) sites on genes located at the 5′ end (*C. velia petG*), in the interior (*C. velia atpB-2*) and 3′ end (*C. velia psbA*) of clusters of related function, as well as on photosynthesis genes that are located within clusters containing genes of otherwise non-photosynthetic function (e.g. *C. velia atpI*, which is positioned downstream of *rps14* and upstream of *rpl11*) [11]. We tested whether poly(U) sites were enriched at the start or end of potential operons in *C. velia*, defining operons as uninterrupted clusters of genes in the genomic sequence that are in the same transcriptional orientation [11], and could not find any significant association (Table S3, chi-squared: P>0.35).

Plant chloroplasts utilise two different RNA polymerases: a nuclear-encoded polymerase related to the phage-type polymerase of mitochondria, which principally transcribes non-photosynthesis genes, and a bacterial-type, plastid-encoded polymerase, principally involved in the expression of photosynthesis genes [34]–[36]. Modulation of the activity of each polymerase may underpin developmental and physiological changes in chloroplast gene expression [37]–[39]. While there is no evidence for the presence of a phage-type plastid polymerase outside the land plant lineage [40], [41], subunits of a bacterial-type polymerase are encoded in the chloroplast genomes of *C. velia and V. brassicaformis* [11]. To test whether this polymerase preferentially transcribes genes that either contain or lack an associated poly(U) site, we searched for predicted bacterial-type promoters across the 5′ UTR of every gene in the *C. velia* chloroplast using a Neural Network Promoter Prediction server [42]. Similar to what has been reported in plants [35], [36], we found evidence for large numbers of candidate promoters in the *C. velia* chloroplast at a wide range of positions, including upstream of photosynthesis and non-photosynthesis genes (Table S3). Across the entire genome, bacterial promoters appeared to be weakly enriched upstream of genes that possess poly(U) sites (chi-squared: P<0.05). However, this was almost entirely due to the fact that bacterial promoters were generally not found upstream of ORFs of unknown function, which are also less likely to possess poly(U) sites than photosynthesis genes. Considering genes of recognisable function, there was no direct association between the presence of predicted bacterial promoters and poly(U) sites (chi-squared: P>0.2). It therefore appears that- at least in the case of a bacterial polymerase- there is not a convincing association between the presence of a candidate promoter and poly(U) site on specific genes.

We additionally analysed the position of poly(U) sites relative to the 3′ end of coding sequences. The position of poly(U) sites relative to the 3′ end of each gene is certainly highly variable, although certain trends were present in each species. Typically, poly(U) sites in *V. brassicaformis* were positioned close to the stop codon, with an average 3′ UTR of 55 nt, although in one case (*petG*) a 3′ UTR of 277 nt was recorded (Table S2). In contrast, poly(U) sites in *C. velia* were positioned further downstream of the stop codon, with an average 3′UTR of 145 nt, and extending up to 584 nt for one poly(U) site found downstream of *psbH* (Table S2). In one case, the *C. velia ORF264* gene, we could identify a poly(U) site that was positioned within the coding sequence itself, 50 bp upstream of the stop codon. This was the only poly(U) site to be found immediately upstream of a predicted termination codon. Oligo-d(A) RT-PCR using a gene specific forward primer, positioned immediately downstream of this poly(U) site, revealed the presence of a second *ORF264* poly(U) site, positioned 281 nt into the 3′ UTR (Tables S1, S2). The sequence covered by the *ORF264* gene does not contain any other sizeable open-reading frames that terminate upstream of the poly(U) site, and it is therefore likely that the addition of a poly(U) tail at the internal site disrupts translation of the *ORF264* transcript.

Several other *C. velia* chloroplast genes appeared to possess multiple potential poly(U) sites, as with *ORF264*. In some instances, oligo-d(A) RT-PCR reactions for *C. velia* produced multiple bands visible after gel electrophoresis (e.g. *atpB-2*; fig. 1, panel A, lane 2; Table S2), which could correspond to multiple alternative poly(U) sites within the 3′ UTR. We assessed variation in poly(U) site position by cloning and sequencing individual RT-PCR products for transcripts from three genes that produced multiple products by oligo-d(A) RT-PCR (*C. velia psaC*, *atpB-2*, *atpI*; Table S4). To identify whether poly(U) sites vary even in genes where no obvious heterogeneity in position could be inferred purely from gel electrophoresis, we cloned and sequenced individual RT-PCR products for *petD* and *psbA* from both *C. velia* and *V. brassicaformis*, each of which produced only a single visible gel band. We found substantial variability in 3′ UTR length for many of the transcripts tested, even if only one band was distinguishable by agarose gel electrophoresis. In one particularly extreme case (*atpB-2*) we observed eleven different poly(U) sites, ranging from 60 to 467 nt into the 3′ UTR (fig. S1, Table S4), which broadly corresponded to the different band sizes visible on oligo-d(A) RT-PCR (fig. 1, panel A, lane 3). In contrast, little variability was seen with *V. brassicaformis petD* and *psbA*, which had consistent tail lengths and only a single nucleotide variability in the 3′UTR prior to the poly(U) tail.

Consistent with this variability, we were unable to identify any conserved sequence motifs located either upstream or downstream of the poly(U) sites in either species. Nor could we identify any consistent changes in GC or purine content, or any predicted secondary structures that were universally associated with poly(U) sites in either species, suggesting that different poly(U) sites might be defined in different ways by the transcript processing machinery. However, ten of the twenty-four poly(U) sites in *V. brassicaformis*, including four sites associated with non-photosynthesis genes (*ccs1*, *chlN*, *rps4*, *rps16*) were immediately adjacent to the 5′ end of predicted tRNAs (Table S2, fig. S2). Although we could identify poly(U) sites that were independent of tRNAs, we could not identify any genes immediately upstream of tRNA genes whose transcripts did not receive poly(U) tails (Table S2). This suggests that some of the poly(U) sites in *V. brassicaformis* are generated by the cleavage of downstream tRNAs from precursor transcripts.

### Poly(U) tail addition is associated with high levels of transcript abundance in *Chromera velia*

It has previously been suggested that the poly(U) tails found in dinoflagellate chloroplasts may facilitate gene expression, either by protecting transcripts from 3′ end degradation [33], or by enabling other transcript processing events, such as editing, that facilitate translation [12], [43]. A recent next generation sequencing study of the *Chromera velia* chloroplast transcriptome, by Janouškovec et al., recovered substantially higher read coverage for transcripts of photosynthesis genes than for transcripts of genes that are not directly involved in photosynthesis, or other open reading frames, suggesting that photosynthesis gene transcripts are highly abundant in the *C. velia* chloroplast [32]. Substantial variation was recorded in transcript abundance within individual operons, indicating that this is at least in part dependent on differences in transcript processing over different genes [32]. We considered whether the presence of a poly(U) tail might be associated with high transcript abundance in chromerid chloroplasts. Calculating from the quantitative read coverage data obtained by Janouškovec et al. [32], genes that possess poly(U) sites are significantly more highly expressed than those that do not (Table S3, Mann-Whitney test, P<E-04).

**Figure 3:**
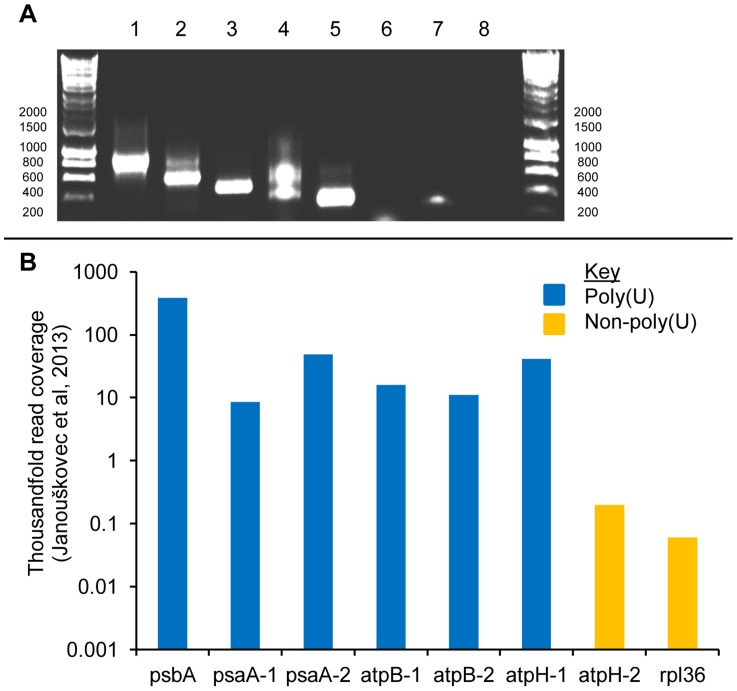
Polyuridylylation of duplicated photosynthesis gene transcripts in the *Chromera velia* chloroplast. This figure shows the result of a series of RT-PCRs to characterise differential patterns of polyuridylylation for different copies of the *psaA*, *atpB*, and *atpH* genes in *C. velia*. Panel A: A gel photo showing oligo-d(A) RT-PCRs for (lanes 1–6) *psaA-1*, *psaA-2*, *atpB-1*, *atpB-2*, *atpH-1* and *atpH-2*. Poly(U) tails were found on transcripts of both *psaA* genes, as has been previously reported, both *atpB* genes, and *atpH-1*, while *atpH-2* was found not to be polyuridylylated. Lanes 7 shows the result of a gene-specific RT-PCR against *atpH-2*, demonstrating that non-polyuridylylated *atpH-2* transcripts are present. Lane 8 shows a template negative control. Panel B: Total abundance of transcripts for each gene in the quantitative read data obtained by Janouškovec et al. [32]. For reference, the most highly expressed (*psbA*) and least abundant *(rpl36)* transcripts encoding recognisable protein-coding function are also shown. Polyuridylylated transcripts are shaded in blue, and non-polyuridylylated transcripts in orange. While all six of the polyuridylylated transcripts shown are highly expressed, *atpH-2* transcripts are found at much lower abundance, only slightly greater than those of *rpl36*, indicating that the *atpH-2* gene may be a pseudogene.

While there may be a general association between polyuridylylation and high levels of expression, other gene-specific factors are also likely to influence transcript abundance, and it is therefore not justifiable to attribute differences in transcript level between different genes solely to the presence of a poly(U) tail. In plant chloroplasts, photosynthesis genes are generally more highly expressed than genes of non-photosynthesis function [44], [45], and the higher transcript abundance associated here with polyuridylylated transcripts, which predominantly function in photosynthesis, could similarly be due to the function of the protein, as opposed to the presence or absence of a poly(U) tail. To gain a more accurate understanding of whether transcript poly(U) tails affect transcript abundance, we considered the expression levels of three photosynthesis genes that are present in the *C. velia* chloroplast genome as multiple copies or fragments. The *psaA* gene is split into two functional units, which encode separate parts of the mature photosystem I reaction centre protein [11], [32]. The two *psaA* transcripts are not trans-spliced together, and are separately translated to form distinct but presumably functionally cooperative proteins [32]. Transcripts of each of the *psaA* genes are highly expressed, and both have been shown to receive a poly(U) tail ([32], fig. 3, panel A, lanes 1–2). A similar situation is true for the *atpB* gene, encoding the chloroplast ATP synthase β subunit, which is likewise split into two functionally autonomous, and highly expressed gene fragments. As with *psaA*, we could detect poly(U) tails on both *atpB* transcripts (fig. 3, panel A, lanes 3–4). In contrast to the *atpB* and *psaA* genes, the *atpH* gene is present in two paralogous copies on the *C. velia* with very different expression patterns. Transcripts of *atpH-1* encode a complete copy of the ATP synthase c subunit, and are highly abundant in chromerid chloroplasts [32]. Transcripts of *atpH-2* encode not only a complete c subunit, but in addition encode a novel 89 aa C-terminal extension not found in protein sequences from other chloroplast lineages (fig. S3, panel A). The *atpH-2* transcripts are nearly one hundredfold lower in abundance than those of *atpH-1*, are the least abundant photosynthesis gene transcripts within the *C. velia* chloroplast, and are only marginally more abundant than *rpl36*, the least abundant transcript of recognisable protein-coding function. This suggests that the *atpH-2* gene is a pseudogene ([32], fig. 3, panel B). The 5′ end and 5′ UTR of the *atpH-2* gene are almost identical to the *atpH-1* gene (93% nucleotide similarity; fig. S3, panel B), suggesting that the difference in transcript abundance is due to sequences within the 3′ extension or 3′ UTR of *atpH-2*. We found that while transcripts of *atpH-1* receive a poly(U) tail, transcripts of *atpH-2* do not (fig. 3, lanes 5–8). The loss of a poly(U) site from the *atpH-2* gene, associated with a much lower level of expression than any other analogous gene in the *C. velia* chloroplast, very strongly indicates that the presence of a poly(U) tail is associated with high levels of gene expression in chromerid chloroplasts.

### Extent of poly(U) addition across chromerid chloroplast transcripts

Given that poly(U) tails appear to be associated with high transcript levels in chromerids, we wished to investigate the precise role of the poly(U) tail in transcript processing. In particular, we wished to determine what proportion of transcripts for individual photosynthesis genes contain poly(U) tails, and whether alternative, poly(U)-independent processing pathways may be present. We accordingly performed RT-PCRs on circularised RNA for a range of chloroplast transcripts in *Chromera velia* using an internal, gene-specific cDNA synthesis primer, an outward-directed PCR reverse primer that annealed just within the 5′ end of coding sequence (CDS), and a PCR forward primer that annealed to the 3′ end of the CDS (Table S1). We tested six genes known to possess poly(U) sites; five photosynthesis genes, of varying levels of transcript abundance (in descending order: *psbA*, *petB*, *psbH*, *atpB(2)*, and *atpI*), and *rps18*, one of only two non-photosynthesis genes found to possess a poly(U) site in both *C. velia* and *Vitrella brassicaformis*. In addition, we tested two genes that lacked an associated poly(U) site: *rps14*, located directly upstream of *atpI*, and *atpH-2*, directly upstream of *psbA*.

**Figure 4:**
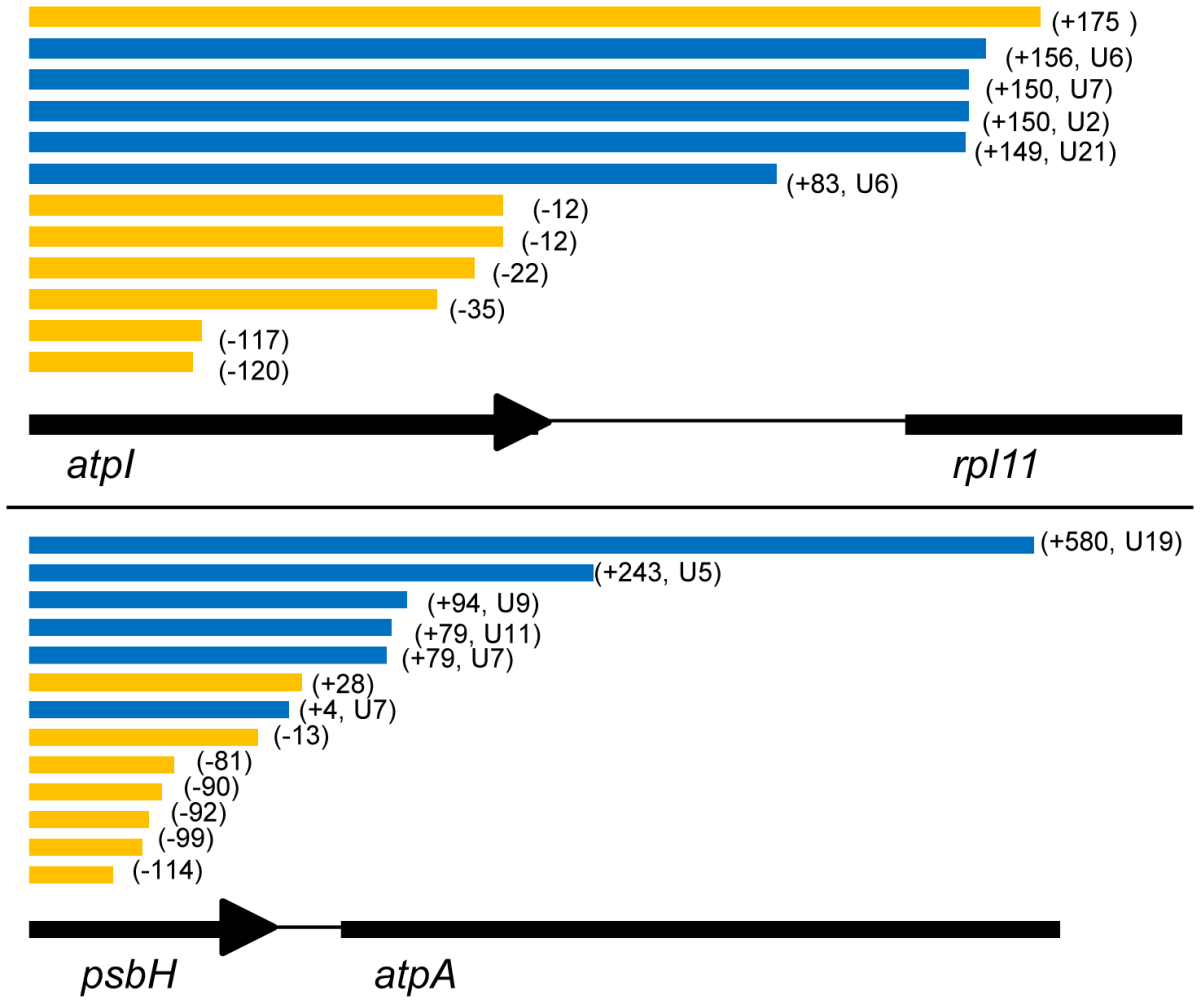
3′ end positions of *atpI* and *psbH* circular RT-PCR sequences. These transcript diagrams show the total number, and polyuridylylation state of transcript 3′ ends mapped for two representative *Chromera velia* genes that had previously been identified to possess poly(U) sites from oligo-d(A) RT-PCR. Circular RT-PCR data for a further six genes is given in Table S5. Transcripts are aligned against the corresponding genomic sequence; polyuridylylated transcripts are shaded in blue, and non-polyuridylylated transcripts in orange. The terminus position of each transcript in the 3′ UTR of the gene is shown in brackets next to the transcript. For both *atpI* (panel A) and *psbH* (panel B), we could obtain several transcripts that terminated in a poly(U) tail, consistent with oligo-d(A) RT-PCR data. We could not identify any other forms of terminal modification on these transcripts. Although we could obtain non-polyuridylylated transcripts for both genes, almost all of these transcripts either terminated within the CDS or upstream of the consensus poly(U) site. Only one non-polyuridylylated *atpI* transcript was found unambiguously to terminate downstream of the consensus *atpI* poly(U) site. It is therefore likely that these non-polyuridylylated transcripts represent the degradation products of previously polyuridylylated transcripts.

We identified homopolymeric poly(U) tails on *C. velia psbA*, *atpB-2*, *atpI*, *petB* and *psbH* transcripts, consistent with the oligo-d(A) RT-PCR data (fig. 4, Table S5, panels A–D). Only two of the polyuridylylated transcripts identified by circular RT-PCR, out of a total of 27 sequenced, contained any nucleotides other than uridine within the 3′ tail, indicating that heteropolymeric tails are extremely rare in chromerid chloroplasts. For each gene, we could additionally identify non-polyuridylylated transcripts, similar to what has been found in dinoflagellates [12], [33], but in almost every case these transcripts terminated either within the CDS or significantly upstream of the poly(U) site in the 3′ UTR of the gene, which may suggest that they are the 3′ degradation products of previously polyuridylylated transcripts (fig. 4). Within the five polyuridylylated photosynthesis genes, we could identify only three transcripts (one transcript each for *psbA*, *petB*, and *atpI*) that terminated at or extended past the corresponding poly(U) sites. Overall, our data are consistent with poly(U) tail addition being the only 3′ maturation pathway acting on photosynthesis gene transcripts.

We could identify transcripts for both *atpH-2* and *rps14* that extended into the 3′ UTR, but could not detect poly(U) tails or any other form of terminal modifications on transcripts of either gene (Table S5, panels A, C). This indicates that transcripts from genes that lack poly(U) sites are not subject to any alternative 3′ modification events. Surprisingly, a circular RT-PCR using primers internal to the *rps18* gene failed to identify any polyuridylylated transcripts (although their existence was indicated by the oligo-d(A) linear RT-PCR experiments), but instead recovered large numbers of transcripts that terminated within the 3′ UTR, upstream of the previously identified consensus poly(U) site (Table S5, panel E). We could retrieve polyuridylylated *rps18* transcripts only by using a PCR forward primer that annealed directly upstream of the *rps18* poly(U) site, thus biasing the PCR for transcripts that extended at least as far as the poly(U) site. However, in this case we also identified equal numbers of transcripts that extended past the consensus poly(U) site (fig. 4, Table S5, panel E). This suggests that the effective concentration of polyuridylylated *rps18* transcripts was very low. Therefore, while *rps18* and some other non-photosynthesis genes may possess poly(U) sites that are detectable by oligo-d(A) RT-PCR, the majority of the corresponding non-photosynthesis gene transcripts do not receive a poly(U) tail during transcript processing. This confirms that the poly(U) tail is principally functionally involved in the processing of photosynthesis gene transcripts in chromerid chloroplasts.

Similar to the variation we observed for transcript 3′ ends, we found that different transcripts identified for a particular gene by circular RT-PCR had different 5′ terminus positions (Table S5). For a few genes, transcripts appeared to terminate preferentially at a certain position within the 5′ UTR: for example, 7 out of 8 *atpH-2* transcripts sequenced terminated 35 nt upstream of the *atpH-2* gene. However, for other genes, we could identify transcripts that terminated at different positions in the 5′ UTR, or terminated at the 5′ end within the CDS (Table S5), suggesting heterogeneous processing of the 5′ end.

### Poly(U) addition is associated with transcript processing in *Chromera velia*

**Figure 5:**
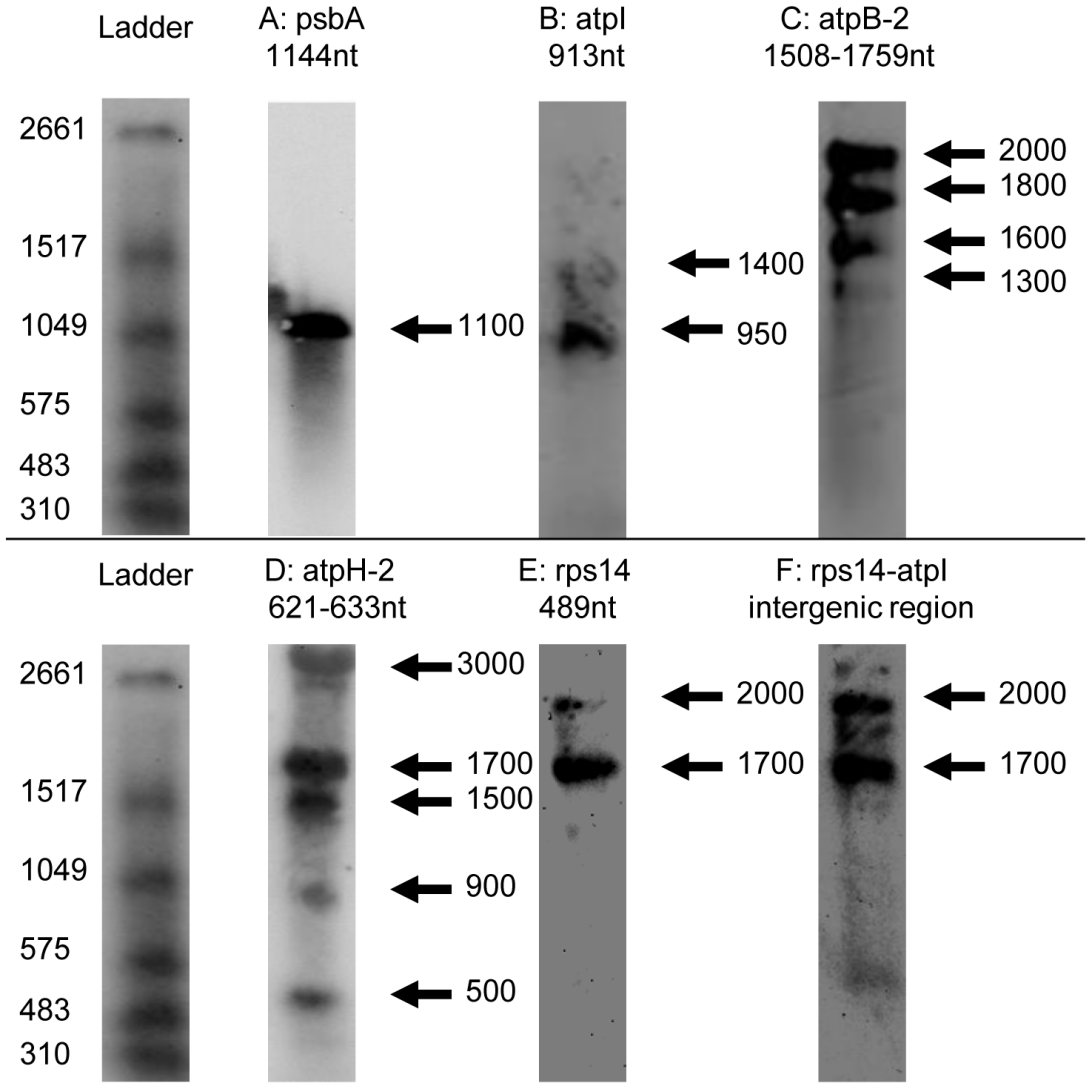
Northern blots of *Chromera velia* chloroplast transcripts. Left, DIG-labelled RNA ladder I (Roche) with approximate size of each band. The sizes of monocistronic polyuridylylated transcripts (panels A-C), or intact monocistronic non-polyuridylylated transcripts (panels D–E) as obtained by circular RT-PCR are listed above the corresponding blot. Panels A–C: northern blots probed for *psbA*, *atpB-2* and *atpI* (all contain an associated poly(U) site). Bands are broadly equivalent to the size of monocistronic transcripts as obtained by circular RT-PCR. Panel D: northern blot probed for *atpH-2*, which lacks an associated poly(U) site. Although a low abundance 500 nt band is present, the most intense bands likely correspond to polycistronic precursors, as obtained by circular RT-PCR, at ≥900 nt. Panel E: *rps14*, which lacks an associated poly(U) site. Bands of an equivalent size to a monocistronic *rps14* transcript are not detectable, and instead, two higher molecular weight bands are observed, at 1700 nt, and at 2000 nt. Panel F: intergenic region between *rps14* and *atpI*, recovering bands of the same size as those in Panel D, indicating that *rps14* transcripts extend through this intergenic region.

It has long been known that chloroplast genes are cotranscribed [46]–[49]. Recently, it has been demonstrated that poly(U) tails are added to polycistronic transcripts in dinoflagellates, indicating that poly(U) tail addition may occur relatively early in transcript processing [15], [33]. We found extensive evidence for polycistronic transcripts in both chromerid species from oligo-d(A) RT-PCR. For some genes for which we could not identify a monocistronic polyuridylylated transcript, polycistronic polyuridylylated products were recovered, with the poly(U) site in the 3′ UTR of the gene furthest downstream. These polycistronic polyuridylylated products extended over two genes (e.g. *C. velia psbK-psbV*) and in one case, even over four genes (*V. brassicaformis rps14-psbV-ccsA-psbK*), (Table S2). At three other selected loci (*C. velia*, *atpH2-psbA*, *ORF207-atpB2*, and *rps14-atpI*), we could specifically amplify transcripts that extended over both genes from oligo-d(A) primed cDNA, using PCR primers that would amplify a region between the 5′ end of the upstream gene and the 3′ end of the downstream gene (fig. S4). To determine whether these polycistronic transcripts are subject to similar 5′ end-processing events as monocistronic transcripts, or uniquely represent primary transcripts, we performed circular RT-PCRs using a primer combination specific to dicistronic *rps14-atpI* transcripts (Table S1, S5). As the T4 RNA ligase used for RNA ligation can only act on transcripts with 5′ monophosphate groups, any products consistent with polycistronic transcripts would indicate that these transcripts had undergone 5′ processing [15], [33]. We could detect a polyuridylylated transcript that extended from the 5′ end of *rps14* to the *atpI* poly(U) site (Table S5, panel B). In total, our data suggest that the chromerid chloroplast transcript pool consists of a mixture of monocistronic, dicistronic and potentially even larger polycistronic transcripts, (U) sites (*psbA*, *atpB-2*, *atpI*; fig. 5, panels A–C), many of which may have undergone 5′ and 3′ end processing.

Given the heterogeneous composition of the chromerid chloroplast transcript pool, we wished to determine how significant a fraction of the *C. velia* chloroplast transcriptome polycistronic transcripts represented. We accordingly analysed northern blots of *C. velia* RNA using probes specific to transcripts of genes that possess poly(U) sites (*psbA, atpI, and atpB-2*), and of genes that do not (*atpH-2*, *rps14*) (Table S6). For each of the genes that possess poly(U) sites, we could identify bands consistent with monocistronic transcripts. For *psbA*, we observed a single band corresponding to an 1100 nt transcript, while for *atpI* we observed a band corresponding to about 950 nt (fig. 5, panels A, B). These agree with expected sizes (from circular RT-PCR results) of monocistronic transcripts (5′ UTR, gene, 3′ UTR and a poly(U) tail) (Table S5). Although we cannot exclude that non-polyuridylylated *psbA* or *atpI* transcripts were also present, we did not identify (by circular RT-PCR) any non-polyuridylylated transcripts, of either gene, of a length that would comigrate with the bands visible in the northern blots (Table S5). For *atpB-2*, we identified multiple bands. The two high intensity bands at 1600 and 1800 nt correspond to the monocistronic, polyuridylylated transcripts obtained by circular RT-PCR (fig. 5, panel C; Table S5). We additionally identified a higher molecular weight band of 2000 nt, and while we could not identify any transcripts by circular RT-PCR of a similar length, this band could plausibly represent monocistronic transcripts that extend to the most distant poly(U) site associated with *atpB-2*, positioned 467 nt into the 3′ UTR (Table S4). No non-polyuridylylated atpB-2 transcripts of 1500 nt length or greater were identified by circular RT-PCR. However, circular RT-PCR (Table S5) did reveal the presence of non-polyuridylylated *atpB-2* transcripts, with 3′ ends internal to the *atpB-2* CDS, which might correspond to a faint 1300 nt band detected in the northern blot (fig. 5, panel C; Table S5).

In contrast to the high abundance of monocistronic transcripts, we could not identify any higher molecular weight bands of a size consistent with polycistronic transcripts, for either *psbA* or *atpB-2* (fig. 5, panel A). We could identify a faint band in the *atpI* northern blot at 1400 nt that might correspond to a polycistronic precursor, but this band was of much lower intensity than the band corresponding to the monocistronic transcript (fig. 5, panel D). Overall, our data indicate that while polycistronic transcripts may be present in chromerid chloroplasts, the transcripts of at least some photosynthesis genes are predominantly present as monocistronic mRNAs, many of which are likely to possess poly(U) tails.

It is possible that the presence of a poly(U) tail might be associated with the processing of polycistronic precursors to monocistronic transcripts. To test this, we probed northern blots for *atpH-2* and *rps14*, which are not polyuridylylated, to determine whether they are processed as efficiently as transcripts of genes that possess poly(U) sites. The *atpH-2* probe sequence was designed against the C-terminal extension unique to the *C. velia atpH-2* gene, and therefore was not expected to cross-hybridise with transcripts of *atpH-1* (Table S6). Surprisingly, a northern blot probed for *atpH-2* recovered several high intensity, high molecular weight bands (fig. 5; panel D). The 900 and 1500 nt bands correspond in size to polycistronic *atpH-2* transcripts obtained by circular RT-PCR that extended well into the *psbA* CDS (Table S5). A lower intensity band at 500 nt was of similar size to degraded transcripts observed in circular RT-PCR that terminated within the *atpH-2* CDS (fig. 5, panel D; Table S5). We could not identify a band of corresponding size (600 nt) to monocistronic *atpH-2*, even though such transcripts were identified using circular RT-PCR. This suggests that monocistronic *atpH-2* transcripts are not present at physiologically significant concentrations (fig. 5, panel D; Table S5).

Similarly, in the case of the *rps14*, which lacks an associated poly(U) site, only bands at 1700 and 2000 nt could be observed, far larger than the c. 500 nt monocistronic transcripts obtained by circular RT-PCR (fig. 5, panel E). The dominant populations of *rps14* transcripts must at least possess a lengthy 3′ UTR, as similar bands were also recovered by a probe that spanned the non-coding region downstream of *rps14* and upstream of the adjacent *atpI* gene (fig. 5; panel F). This implies that, at least at certain loci, transcripts of genes that lack associated poly(U) sites may be subject to limited 3′ end-maturation events, and are instead retained on higher molecular weight precursors.

### Transcripts in the *C. velia* chloroplast are subject to alternative processing

For several of the oligo-d(A) RT-PCR products sequenced (e.g. *C. velia atpI*, *V. brassicaformis rps18*), the poly(U) site associated with a particular gene is positioned within the 5′ end of the downstream coding sequence. If these polyuridylylated transcripts are generated from the cleavage of longer, polycistronic precursors, the poly(U) sites would indicate that transcripts are generated by alternative 3′ processing, in that the generation of a mature mRNA from a polycistronic precursor transcript would prevent the generation of an mRNA of the downstream gene from the same precursor. Such alternative processing is similar to what has been previously identified in other chloroplast lineages [33], [47].

To determine whether alternative processing can occur in chromerid chloroplasts, we investigated transcript processing at the *C. velia petG-petB-psbH-atpA* locus (fig. S5). We identified polyuridylylated dicistronic *petG-petB* and *petB-psbH* transcripts, using similar RT-PCRs as before (fig. S5), demonstrating that polyuridylylated polycistronic transcripts are present over this locus. The poly(U) site associated with *petB* is located 27 nt within the 5′ end of *psbH*, and the *psbH* poly(U) site is located up to 584 nt into *atpA* (Table S2), hence it would be impossible to generate complete *psbH* transcripts from a precursor that had already yielded a polyuridylylated *petB* transcript, or *atpA* transcripts from a precursor that yielded *psbH*. We could additionally identify polyuridylylated monocistronic *petB*, monocistronic *psbH*, and dicistronic *petB-psbH* transcripts by circular RT-PCR (Table S5, panel D). This indicates that transcripts at this locus do undergo some degree of 5′ cleavage, and that monocistronic transcripts could plausibly be cleaved from polycistronic precursors.

**Figure 6:**
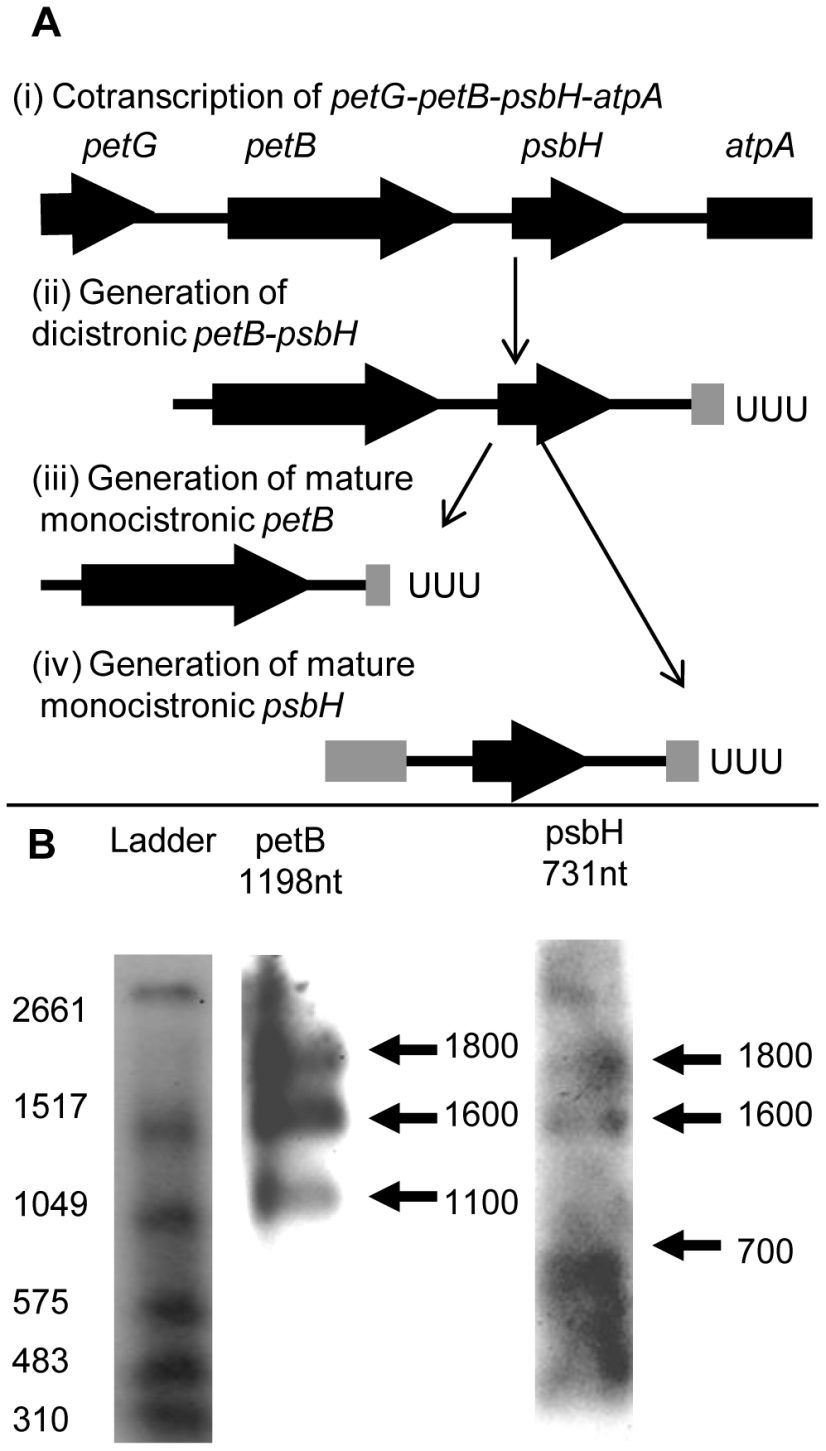
Processing of *Chromera velia* petB and psbH transcripts. Panel A shows one possible model for the alternative processing of transcripts over the *C. velia petB-psbH* locus. Complete coding sequences are shown by thick black arrows, and non-coding DNA by thin black lines. Thick grey lines show incomplete regions of coding sequence on transcript ends that have been generated by alternative processing. Vertical arrows show the likely progression of transcript processing events. The *petB* and *psbH* genes are initially cotranscribed from a promoter element located upstream of the *petB* gene, as part of a long polycistronic transcript that may also extend over the *petG* and *atpA* genes (i). The initial primary transcript generated is likely to be processed at both the 5′ and 3′ ends to form shorter polycistronic intermediates, such as a dicistronic polyuridylylated *petB-psbH* transcript that extends from the *petB* 5′ UTR to a poly(U) site positioned downstream of *psbH*, within the *atpA* CDS (ii). This dicistronic transcript may then be cleaved to form either a monocistronic polyuridylylated *petB* (iii) or *psbH* transcript (iv). As the *petB* poly(U) site is positioned within the *psbH* CDS (iii), and the *psbH* 5′ end is positioned within the *petB* CDS (iv), mature, monocistronic *petB* and *psbH* transcripts cannot be generated from the same precursor, and thus are cleaved from different transcript precursors via mutually exclusive processing steps. Panel B shows northern blots analysed using probes against the *petB* and *psbH* genes. Left DIG-labelled RNA ladder I (Roche) with sizes indicated. The sizes of monocistronic polyuridylylated transcripts as obtained by circular RT-PCR are listed above the corresponding blot. In each blot, two conserved higher molecular weight bands are present at 1600 and 1800 nt, which are likely to likely represent polycistronic precursors covering the *petB* and *psbH* coding sequences. In addition, lower molecular weight bands unique to either the *petB* (1100 nt) or *psbH* blots are observed (700 nt), consistent with monocistronic transcripts as recovered by circular RT-PCR.

It is possible that, instead of being generated by the processing of common, polycistronic precursors, mRNAs in chromerid chloroplasts that have overlapping terminus regions might be separately transcribed from different promoter sites in the 5′UTR of each gene, and accumulate as independent populations of transcripts. Although we cannot exclude the possibility that some *psbH* transcripts are independently transcribed from promoter sequences promoter elements positioned between the *petB* and *psbH* genes, we could identify *psbH* transcripts from circular RT-PCR that extend at the 5′ end into the *petB* CDS by up to 261 nt (Table S5, panel D), which clearly must have been initiated from elements further upstream. While it is possible that *psbH* transcripts are generated from a promoter internal to the *petB* CDS, internal promoter sites are uncommon at least for protein-coding genes in plant chloroplasts, and generally appear to give rise only to very low levels of transcripts [35], [36], [50], so it is unlikely that the transcriptional products of an internal promoter sequence would be detectable by circular RT-PCR. Taken together, our data therefore indicates that *petB* and *psbH* transcripts are most likely to be cotranscribed from a common promoter element upstream of the *petB* 5′ end. In at least some cases, mature, monocistronic *petB* and *psbH* mRNAs are generated by the alternative 5′ and 3′ cleavage of a common polycistronic precursor, which may be the dicistronic *petB-psbH* transcripts obtained by circular RT-PCR (fig. 6, panel A).

Finally, we wished to determine whether alternatively processed transcripts comprise a significant proportion of the chromerid chloroplast transcript pool. We investigated the relative abundance of different processing intermediates over the *petB-psbH* locus, by analysing northern blots with probes for the *C. velia petB* and *psbH* genes (fig. 6). The *psbH* probe was positioned downstream of the *petB* poly(U) site, and the *petB* probe was positioned so that there was minimal overlap with the 5′ end of *psbH* transcripts (Table S1, S5, S6). Monocistronic transcripts should therefore only be detectable in either the *petB* or *psbH* blots, whereas common polycistronic precursors should be detectable in both. We observed 1600 and 1800 nt bands in both the *petB* and *psbH* blots (fig. 6, panel B). The 1600 nt band was of an equivalent size to polyuridylylated, dicistronic *petB-psbH* transcripts obtained by circular RT-PCR (Table S5, panel D), indicating that polycistronic transcripts are abundant over this locus. We could not identify any other high molecular weight bands of high intensity in either blot. However, we also observed lower molecular weight bands that were specific to either the *petB* or the *psbH* blots (fig. 6, panel B). The 1100 nt band seen when probed for *petB* is similar in size to a monocistronic transcript, which terminates at the 5′ end in the intergenic region between *petG* and *petB*, and at the 3′ end in the *psbH* CDS, as obtained by circular RT-PCR (fig. 6, panel B; Table S5, panel D). Similarly, the 700 nt band seen when probed for *psbH* is similar in size to the circular RT-PCR sequence of a monocistronic transcript that terminates at the 5′ end in the *petB* CDS, and at the 3′ end in the *atpA* CDS (fig. 6, panel B; Table S5, panel D). This indicates that monocistronic *petB* and *psbH* mRNAs, generated by alternative processing, do accumulate to a detectable level in *C. velia* chloroplasts. If poly(U) tails are added to transcripts prior to 5′ processing, the selection of a specific poly(U) site may even be involved in specifying which 5′ end maturation pathway occurs.

### *Plasmodium falciparum* apicoplast transcripts are not polyuridylylated

**Figure 7:**
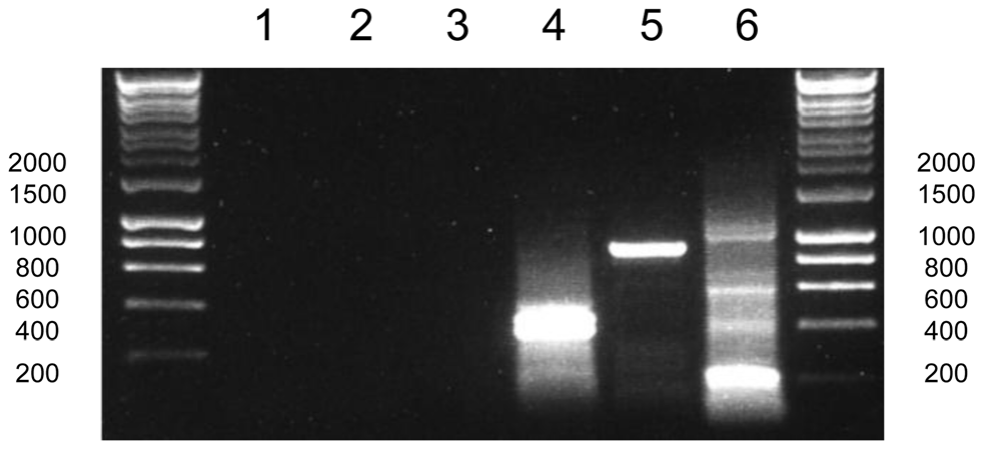
Poly(U) tails are not added to apicoplast transcripts in *Plasmodium falciparum*. This gel photo shows the result of a series of RT-PCRs to characterise polyuridylylation for transcripts from the *P. falciparum rpoB*, *rpoC* and *sufB* genes. Lanes 1–3: Oligo-d(A) RT-PCRs for *rpoC* (lane 1), *rpoD* (lane 2) and *sufB* (lane 3) transcripts. Lanes 4–6: Gene-specific RT-PCR for *rpoC*, *rpoD* and *sufB*.

As transcripts from non-photosynthesis genes in chromerid chloroplasts are generally not polyuridylylated, we wished to determine whether poly(U) tails were added to apicoplast transcripts, since proteins encoded in the apicoplast genome do not function in photosynthesis. We accordingly performed oligo-d(A) RT-PCRs as before, using RNA from *Plasmodium falciparum*, and PCR forward primers specific to all thirty protein-coding genes in the apicoplast. Oligo-d(A) RT-PCRs against apicoplast genes typically did not yield distinct bands (Fig. 7, lanes 1–3). In some cases, we could observe faint bands on gel electrophoresis of oligo-d(A) RT-PCR products, which could potentially represent polyuridylylated transcripts. However, when sequenced, no product corresponded to apicoplast sequences. In contrast, we could identify non-polyuridylylated transcripts for apicoplast genes by amplification of cDNA generated using gene-specific primers (fig. 7, lanes 4–6). We therefore conclude that only non-polyuridylylated transcripts are present in the *P. falciparum* apicoplast.

## Discussion

We have characterised the distribution and function of poly(U) sites across the chloroplast genomes of the chromerid algae *Chromera velia* and *Vitrella brassicaformis*, and have shown that *Plasmodium falciparum* transcripts do not undergo polyuridylylation. The poly(U) sites found on chromerid chloroplast transcripts share some degree of similarity with those of dinoflagellates. Variable or alternative poly(U) sites, which appear to be particularly widespread in *C. velia*, have also been observed in several dinoflagellate species [12], [16], [33], [51]. Furthermore, the association between poly(U) sites and tRNA cleavage in *V. brassicaformis* has previously been suggested for the dinoflagellate *Heterocapsa triquetra* [43], [51]. However, unlike in dinoflagellates, polyuridylylation occurs only on specific transcripts in chromerid chloroplasts. To date, only one protein-coding gene that lacks an associated poly(U) site has been identified in a peridinin dinoflagellate chloroplast - *petD* in *Amphidinium carterae* [15]. Conversely, large numbers of protein-coding genes in both the *C. velia* and *V. brassicaformis* chloroplasts lack an associated poly(U) site, and these principally encode products that do not directly function in photosynthetic electron transfer. While we could identify a small number of photosynthesis genes in either *C. velia* or *V. brassicaformis* that lacked poly(U) sites, or transcripts of non-photosynthesis genes that were polyuridylylated (Table S2, fig. 2), very few of these exceptions were conserved between both species, and our *rps18* circular RT-PCR data suggest that at least some of the poly(U) sites associated with non-photosynthesis genes may not be physiologically significant (Table S5, panel E). Thus, the polyuridylylation of chromerid chloroplast transcripts appears to largely be dependent on a photosynthetic function of the translation product.

With this in mind, the function of transcript polyuridylylation in chromerid chloroplasts is particularly intriguing. The high expression level of photosynthesis gene transcripts, which has been suggested to help enable rapid photo-physiological adaptation to changing light conditions [27], [32], may suggest that the poly(U) tail facilitates transcript accumulation in chromerid chloroplasts. Transcript processing complexes are known to be involved in negative regulation of non-coding transcripts in other organelle lineages [4], [52]. The presence or absence of a poly(U) site might similarly be involved in discriminating between functional and non-functional photosynthesis gene transcripts, such as those of the functional *atpH-1* gene and the non-functional *atpH-2* gene, and might potentially determine the levels to which they accumulate in chromerid chloroplasts (fig. 3). Notably, the *atpH-2* CDS itself does not contain in-frame premature termination codons, or any other features that would directly prevent its expression. Thus, the loss of a poly(U) site on the *atpH-2* transcript, and consequent reduction in transcript abundance [32], could minimise expression of *atpH-2* without inactivation of the underlying gene sequence. It remains to be determined whether atpH-2 protein accumulates to a significant level in *C. velia* chloroplasts, but our data as a whole certainly suggest that poly(U) tails may facilitate expression of functional copies of photosynthesis genes in chromerid chloroplasts.

One possible means by which the poly(U) tail could facilitate gene expression is by coordinating other chloroplast transcript processing events. As polyuridylylated polycistronic transcripts are present in chromerid chloroplasts, poly(U) tails might be added relatively early in transcript processing. For certain genes that possess poly(U) sites (*petB*, *psbH*), polycistronic transcripts accumulate to concentrations detectable in northern blots, but in these and other genes (e.g. *psbA*, *atpB-2*, *atpI*), monocistronic mRNAs, which have presumably been cleaved from polycistronic precursors, are abundant (fig. 5, panels A–C, fig. 6). Similar patterns of transcript abundance have recently been reported for other photosynthesis genes in *C. velia* by Janouškovec et al. [32]. In contrast to the high levels of transcript processing observed for polyuridylylated genes, transcripts from the *rps14* and *atpH-2* genes (which do not contain associated poly(U) sites) are predominantly present as high molecular-weight precursors (fig. 5, panels D–F), indicating that transcripts are subject to very limited 3′ end processing in the absence of a poly(U) tail, and the presence of a poly(U) site could be associated with the cleavage of polycistronic transcripts to monocistronic mRNAs. At loci such as *C. velia petG-petB-psbH* that contain multiple possible poly(U) sites, the selection of a poly(U) site may define which products are generated. Alternative processing of precursors containing multiple potential photosynthesis gene transcripts has previously been suggested to occur in dinoflagellates [15], [33], and is similar to alternative 3′ polyadenylylation sites previously observed in nuclear genomes, which may substantially alter the coding capacity and regulatory properties of nuclear transcripts [53], [54]. It will be interesting to test how the presence of a poly(U) tail may influence the accumulation and expression of polyuridylylated transcripts. For example, polyuridylylated transcripts might be more stable than non-polyuridylylated transcripts following the inhibition of chloroplast transcription, or be more frequently associated with polysomal fractions in chromerid chloroplasts..

**Figure 8:**
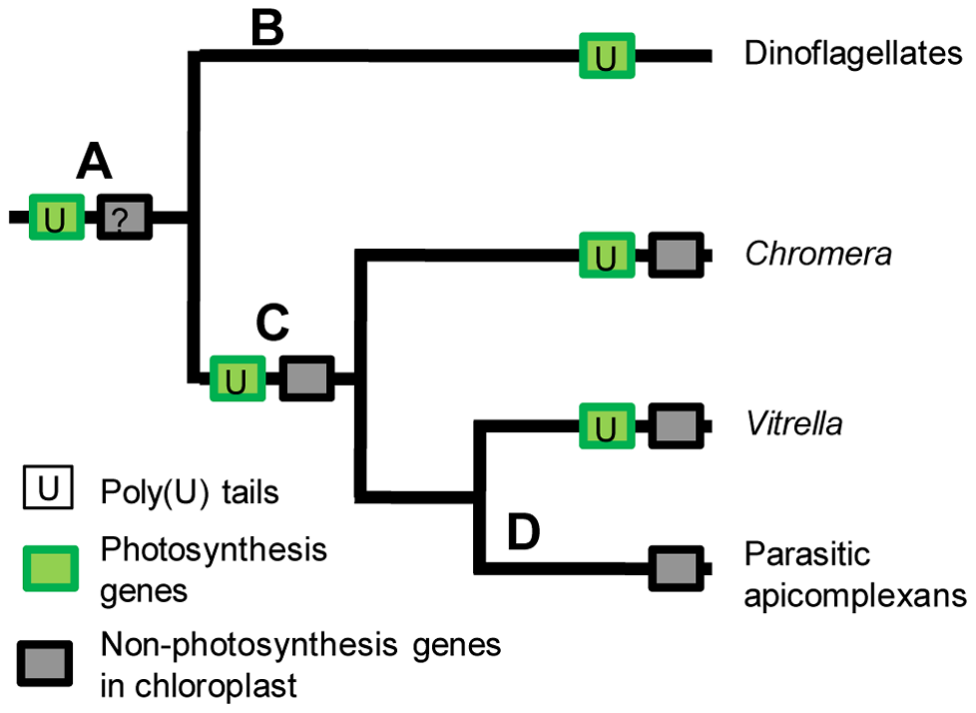
The evolution of transcript polyuridylylation in the alveolates. This schematic figure shows the evolutionary history of the chloroplasts of dinoflagellates, apicomplexans, and chromerids . The phylogenetic relationships between each lineage are given as per [11]. The content of each associated chloroplast genome is depicted by shaded boxes on each branch. The polyuridylylation of chloroplast transcripts is likely to have arisen once, in a common ancestor of all four lineages, which contained both photosynthesis and non-photosynthesis genes in its genome (point A). Following their divergence from the other three lineages, dinoflagellates relocated all non-photosynthesis genes to the nucleus, leaving only photosynthesis genes, of which almost all possess an associated poly(U) site (point B). As a result, it is not possible to determine whether the chloroplast polyuridylylation machinery was initially applied to all chloroplast transcripts, or only to photosynthesis gene transcripts; however, the polyuridylylation machinery of the last common ancestor of chromerids and apicomplexans was certainly specific to photosynthesis genes (point C). While this situation has broadly remained true in each chromerid lineage, the apicomplexans have subsequently lost both photosynthesis genes and the associated chloroplast transcript processing machinery from their chloroplast (point D).

The distribution and function of the poly(U) machinery in chromerid chloroplasts may underline key events in the evolution of the non-photosynthetic apicoplast found in apicomplexans. The most parsimonious scenario is that transcript polyuridylylation arose in a photosynthetic common ancestor of chromerids, dinoflagellates, and apicomplexans (fig. 8, point A). It is not possible to determine whether poly(U) tails in this common ancestor were added only to photosynthesis transcripts, or were initially applied to all chloroplast transcripts with specificity arising later, as the chloroplasts of peridinin dinoflagellates do not retain any recognisable genes of non-photosynthetic function, which instead have been almost entirely relocated to the nucleus [13], [55], [56] (fig. 8, point B). However, in the common ancestor of chromerids and apicomplexans, the polyuridylylation machinery exclusively targeted transcripts of photosynthesis genes (fig. 8, point C), with specific exceptions and counterexamples having arisen subsequently in each lineage since their divergence. In each chromerid species, poly(U) sites have been lost from a small number of photosynthesis genes, and gained by a few non-photosynthesis genes. In contrast, within parasitic apicomplexans, all of the photosynthesis genes have been lost from the apicoplast, presumably concomitantly with the loss of the associated polyuridylylation machinery (fig. 8, point D) [10]. It is possible that an early ancestor of apicomplexans changed from a photosynthetic to a non-photosynthetic lifestyle, and the poly(U) machinery was subsequently lost due to a lack of selective pressure for its retention. Equally, if the presence of a poly(U) pathway were essential for the correct processing of photosynthesis gene transcripts, then the loss of the protein(s) involved in polyuridylylation might have been a key step in the transition from a photosynthetic to a parasitic lifestyle. Examples are known from parasitic plants where the loss of a consensus transcript processing site appears to precede inactivation of a chloroplast gene, and presumably its eventual loss of the chloroplast genome [4], [57]. Further analysis of the gene expression machinery of chromerids may provide important insights into the evolutionary steps required to convert a photosynthetic alga into a non-photosynthetic parasite such as *Plasmodium*.

## Materials and Methods

### Ethics statement

All work involving human blood was carried out in accordance with the UK Human Tissue Act (2004), and we thank our anonymous donors for their blood.

### Cultures

Liquid cultures of *Chromera velia* CCMP2878 were grown in f/2 medium at 18°C, under 30 µE illumination on a 16∶8 h L∶D cycle. Cultures were harvested at 18 days post-inoculation (mid-log phase) for RT-PCR, and at 30 days (early stationary phase) for northern blotting. *C. velia* cells were predominantly in coccoid form at all time points harvested.

*Vitrella brassicaformis* CCMP3155 were grown under the same conditions in f/2 medium supplemented with 100 µg/l ampicillin, and 20 µg/l each kanamycin and spectinomycin. Cultures were harvested at approximately two to three months post-inoculation, at which point pigmented colonies of vegetative cells were visible in the culture flask. *Amphidinium carterae* CCMP 1314 and *Phaeodactylum tricornutum* CCAP 1055/2 were grown under the same conditions in f/2 medium as previously described [12], and harvested at 25 days post-inoculation (mid-log phase).

*Plasmodium falciparum* was cultured in donated red blood cells according to a method previously described [58].

### Nucleic acid isolation

Mature cultures of *C. velia* and *V. brassicaformis* were pelleted, washed twice with sterile artificial seawater, and resuspended in 1 ml Trizol reagent (Invitrogen)/30 mg cells. Each resuspension was ground to a powder in liquid nitrogen in a clean pestle and mortar that had been prewashed in 10% hydrogen peroxide to remove RNase.

The powdered cells were resuspended in an additional 1 ml Trizol/30 mg cells, and Trizol phase extraction, DNase treatment and RNA cleanup was performed as previously described [12], [33]. Each RNA sample was confirmed to be DNA-free through two rounds of direct PCR. Total RNA was harvested from asynchronous *P. falciparum* culture using Trizol (Invitrogen) as previously described [59]. RNA for use in RT-PCR reactions was stored at −80°C in diethylpyrocarbonate-treated water. RNA for use in northern blots was not subjected to DNase treatment, but resuspended immediately following precipitation in formamide, and stored at −80°C. Genomic DNA was harvested from *C. velia* as has previously been described [11].

### RT-PCR and sequencing

Oligo-d(A) and gene-specific RT-PCRs were performed for *C. velia* and *V. brassicaformis* as previously described [12]. Due to the extreme AT-richness of the genome, *P. falciparum* PCR reactions were carried out for 30 elongation cycles, with an annealing temperature of 50°C and an extension temperature of 60°C. RNA of *C. velia* was circularised, and circular RT-PCRs were performed using previously described methods [12], [33]. Primers for each RT-PCR reaction are tabulated in Table S4.

PCR products were purified using the MinElute PCR cleanup kit (Qiagen). Cloned products were ligated into pGEM-T Easy vector (Promega), transformed into competent *Escherichia coli* DH5α, and purified with either a GeneJET miniprep kit (Fermentas) or a Qiagen miniprep kit prior to sequencing. Products were sequenced using an Applied Biosystems 3730xl DNA Analyzer.

### Sequence analysis

Sequences were aligned against the *C. velia* and *V. brassicaformis* chloroplast genome using MAFFT (http://mafft.cbrc.jp/alignment/software/). To identify putative bacterial promoters in the *C. velia* chloroplast, we extracted the 5′ UTR sequence of each gene, and searched for promoter sequences using the Neural Network Promoter Prediction server [42] (http://www.fruitfly.org/seq_tools/promoter.html). A pilot experiment was performed using the barley chloroplast genome, for which promoters have been extensively characterised [36] , and a cut-off value of 0.8 was selected as identifying the highest number of promoters with a minimal false positive rate.

To identify putative sequences associated with poly(U) sites, alignments of every 3′ UTR sequence, and the 100 bp of genomic sequence downstream of each poly(U) site, were constructed. To search for sequences with conserved patterns of purines and pyrimidines, sequences were manually recoded using RY IUPAC nomenclature, as has previously been described [60]. Conserved primary sequences were searched by visual inspection of alignments, by reciprocal BLASTing of each sequence against each other sequence within the alignment, and with three online motif search programs: Bioprospector (http://robotics.stanford.edu/~xsliu/BioProspector/), Improbizer(http://users.soe.ucsc.edu/~kent/improbizer/improbizer.html), and PhyloGibbs(http://www.phylogibbs.unibas.ch/cgi-bin/phylogibbs.pl). GC contents were plotted using the crude alignments and GeneIOUS Pro (http://www.geneious.com/). Conserved secondary structures were searched using the locARNA (http://www.bioinf.uni-freiburg.de/Software/LocARNA/) and Carnac web servers (http://bioinfo.lifl.fr/carnac/). Oligo-d(A) RT-PCR products were deposited in GenBank (Accession numbers KC618536-KC618583).

### Northern blotting

Northern blots were performed using a DIG northern starter kit (Roche). For each blot, 3 µg total cellular RNA was diluted to 20 µl in formamide, melted at 65°C for 5 minutes, snap frozen, and separated by electrophoresis at 100 V on a 1% TBE-agarose gel, containing 500 mg/l guanidine thiocyanate, for 90 minutes. 4 µl DIG-labelled RNA ladder I (Roche), again diluted to 20 µl in formamide, melted and snap frozen, run alongside as a size marker, and a formamide-only lane was run as a negative control. The northern transfer was set up overnight per the manufacturer's instructions, using a positively charged nitrocellulose membrane (Roche). To confirm RNA integrity during electrophoresis, an additional lane of total cellular RNA was run out, stained post-hoc in ethidium bromide, and visualised with UV. The residual gel slices left following compression were likewise stained and visualised to confirm RNA integrity during transfer.

Blots were hybridised overnight at 65°C against complementary RNA probes, generated by in vitro transcription of a template sequence, using T7 RNA polymerase and digoxigenin-labelled nucleotides. Template sequences were generated by ligating PCR products against desired regions of the *C. velia* chloroplast genome into pGEM-T Easy vector sequence (Promega), and amplifying the ligation products using a T7 primer and a PCR forward primer, to generate products containing the short, 49 bp T7 arm of the vector sequence fused to an antisense orientation insert. Probe sequences are tabulated in Table S1. Hybridisation products were visualised using an anti-digoxigenin/CPD-star system (Roche), per the manufacturer's instructions.

## Supporting Information

Figure S1 Associated poly(U) sites of the *Chromera velia atpB-2* gene. This alignment shows the first 500 bp downstream of the *Chromera velia atpB-2* gene. Grey arrows correspond to the different poly(U) sites, identified from the sequences of twenty randomly selected separate, individual cloned oligo-d(A) RT-PCR products using a gene-specific forward PCR primer against *C. velia atpB-2*. Numbers indicate that multiple colonies gave rise to the same poly(U) site.
(TIFF)

Figure S2 tRNA-associated poly(U) sites in *Vitrella brassicaformis*. These diagrams show the 3′ UTRs of transcripts for the *V. brassicaformis psaD* and ccs1 genes, as defined by oligo-d(A) RT-PCR. Grey arrows show the associated poly(U) addition sites for each transcript; the poly(U) tail is not directly shown. In both genes, the poly(U) site is positioned immediately upstream of an associated tRNA (respectively tRNL-CAA and tRNN-GUU). The position and structure of each tRNA, as predicted by the tRNAscan-SE server (http://lowelab.ucsc.edu/tRNAscan-SE/) is shown for each transcript sequence.
(TIFF)

Figure S3 Alignments of chromerid chloroplast atpH sequences. Panel A shows a protein alignment contains the predicted translation products of *Chromera velia atpH-1* and *atpH-2*, and *Vitrella brassicaformis atpH*, as well as sequences from other representative photosynthetic eukaryotes. Sequence alignments were constructed using MAFFT (http://mafft.cbrc.jp/alignment/server/index.html) using the default settings. An 85% consensus sequence is given at the top of the alignment; characters that match the consensus are shaded for each sequence. The predicted translation product of *C. velia atpH-2* contains an 89aa C-terminal extension not found in any other AtpH sequence. Panel B shows nucleotide sequence BLAST alignments for the 5′ end of the coding sequence (i) and 5′ UTR (ii) of the *C. velia atpH* gene copies. For each alignment, *atpH-1* is shown in the query line, and *atpH-2* in the subject line. A high degree of sequence conservation (93%) is observed for both regions. This suggests that the very different transcript abundances observed for each gene copy is likely to be dependent on sequence features at the 3′ end of each transcript.
(TIFF)

Figure S4 Polycistronic polyuridylylated transcripts in *Chromera velia*. *atpH2-psbA*, *ORF247-atpB2*, and *rps14-atpI* all consist of an upstream gene that lacks an associated poly(U) site, and a downstream gene that contains an associated poly(U) site as shown in the diagram. Oligo-d(A) cDNA was used as the PCR template, and a PCR was performed to identify dicistronic transcripts, using a forward primer against the 5′ end of the upstream gene, and a reverse primer internal to the downstream gene. PCR over the *atpH2-psbA* intergenic region using lane 1: oligo-d(A) cDNA; lane 2, gDNA; lane 3, template negative conditions. Lanes 4–6: as lanes 1–3 with *ORF247-atpB2* locus. Lanes 7–9: as lanes 1–3 with *rps14-atpI* locus.
(TIFF)

Figure S5 Cotranscription of the *Chromera velia petG-petB-psbH* locus. As figs. 3 and 4, a transcript diagram with each of the PCR amplicons tested is shown beneath the gel photo. Lanes 1–3: oligo-d(A) RT-PCR for *psbH*, *petB* and *petG* transcripts (all polyuridylylated). The poly(U) sites associated with the *petB* and *psbH* genes are positioned respectively inside the 5′ ends of the *psbH* and *atpA* coding sequences, hence mature *petB*, *psbH* and *atpA* mRNAs cannot be generated from the same transcript. lanes 4–5: oligo-d(A) RT-PCR for the intergenic *petG-petB* and *petB-psbH* regions; lanes 6–7: PCR for the same intergenic regions using DNA template; lanes 8–9: PCR for the same intergenic region using template negative conditions. The positive results for lanes 4–5 indicates that individual poly(U) sites within this locus are generated by alternative 3′ processing of polycistronic precursors.
(TIFF)

Table S1 Tabulated primers used. Panel 1 gives primers for oligo-d(A) RT-PCRs of chromerid chloroplast transcripts (including control reactions) and nested RT-PCRs to detect dicistronic polyuridylylated transcripts in *Chromera velia*, and Panel 2 gives primers used for circular RT-PCRs. Panel 3 gives primers for oligo-d(A) RT-PCRs of the *Plasmodium falciparum* apicoplast.
(XLSX)

Table S2 Tabulated results for oligo-d(A) RT-PCRs against *Chromera velia* and *Vitrella brassicaformis* chloroplast transcripts. This table shows the result of every diagnostic oligo-d(A) RT-PCR reaction performed for chromerid chloroplast genes in this study. Each studied gene is listed either as having a direct function in photosynthetic electron transfer (PS), as having a defined function that is not directly associated with photosynthesis (NON-PS), or as being an ORF with no defined function (ORF). The results of oligo-d(A) RT-PCRs are given as follows: (Y), a poly(U) site is associated with a given gene, as confirmed by direct sequencing of the oligo-d(A) RT-PCR product; (−), there is no poly(U) site associated with the given gene, as confirmed by negative oligo-d(A) primed RT-PCR results through two successive rounds of PCR amplification; (n/a), the gene is absent from the given chloroplast genome. The positions of each poly(U) site on the NCBI accessions for each chromerid chloroplast genome sequence are given. Genes that are asterisked were found to have multiple alternative associated poly(U) sites either from the presence of multiple bands on oligo-d(A) RT-PCR gels, or through cloning and sequencing of individual RT-PCR products; here, the most extreme positions and values identified are given. The total frequency of polyuridylylated photosynthesis and non-photosynthesis genes, the mean 3′ UTR length, and the mean poly(U) tail length for each species are given at the bottom. In genes where multiple potential poly(U) sites were identified, the averages of the most extreme values observed were used for calculations of total species mean values.
(XLSX)

Table S3 Bioinformatic analysis of the distribution of poly(U) sites in *Chromera velia*. Panel 1 tabulates the polyuridylylation state, predicted function, and location of each gene within operons, and the presence of predicted bacterial promoters in the *C. velia* chloroplast genome. Operons are defined from the genomic sequence [11], and promoter sites are predicted via a Neural Network prediction server [42]. The chi-squared association values against poly(U) sites for each value are shown at the bottom, calculated both against the genome as a whole, and against genes of recognisable predicted function only. Panel 2 gives an exemplar list of bacterial-type promoters obtained by screening the first 50 kbp of the *C. velia* genome, with a threshold probability of 0.8. Panel 3 tabulates the polyuridylylation state of each gene against the mean read coverage obtained by Janouškovec et al. [32], in absolute terms, logarithmic terms, and ranked terms. The mean transcript abundance for genes that possess poly(U) sites versus genes that do not is shown below, calculating against the genome as a whole, and against each functional category of genes (photosynthesis genes, non-photosynthesis genes, and unannotated ORFs). In each case tested, genes that possess poly(U) sites are more abundant represented in the *C. velia* chloroplast transcript pool than genes that lack poly(U) sites.
(XLSX)

Table S4 Alternative poly(U) sites observed in chromerid chloroplast genes. This table shows each of the poly(U) sites observed by cloning and sequencing individual oligo-d(A) RT-PCR products for the *Chromera velia atpB-2*, *atpI*, *petD*, *psaC* and *psbA* genes, and the *petD* and *psbA* genes of *Vitrella brassicaformis*. As per Table S2, the position of the poly(U) site on the corresponding genome sequence, plus the 3′ UTR and poly(U) tail lengths are given.
(XLSX)

Table S5 Tabulated circular RT-PCR sequences for *Chromera velia psbA,atpH-2*, *atpB-2*, *atpI*, *rps14*, *petB*, *psbH* and *rps18*. These tables show the terminus positions of the eight genes studied by circular RT-PCR, and the transcript sequences obtained for them. Transcripts are grouped by the primer combinations used to obtain them. For *rps18*, two alternative PCR forward primers were used: a primer that annealed within the 3′ end of the CDS (series A), and a primer that annealed immediately upstream of the poly(U) site, within the 2′ UTR (series B). For each transcript, the start and end positions on the genomic sequence is shown, as well as the transcript length, and the distance of the 5′ and 3′ ends from the gene termini. Unless specifically noted otherwise, non-polyuridylylated transcripts terminate upstream of the consensus poly(U) site for the gene. Transcripts of equivalent size to bands detected in the corresponding northern blots (figs. 5, 6) are highlighted in bold.
(XLSX)

Table S6 Tabulated probes used for northern blots of *Chromera velia* chloroplast transcripts. For each probe, both the sequence itself, including the T7 arm of the pGEM-tEasy vector, and the positions on the *C. velia* chloroplast genome from which the probe was generated are given.
(XLSX)
