# Supplementary figures and images for: Evolution of Chloroplast Transcript Processing in Plasmodium and Its Chromerid Algal Relatives
Source: PLoS Genet. 2014 Jan 16;10(1):e1004008. doi: 10.1371/journal.pgen.1004008 (PMC3894158; doi:10.1371/journal.pgen.1004008)

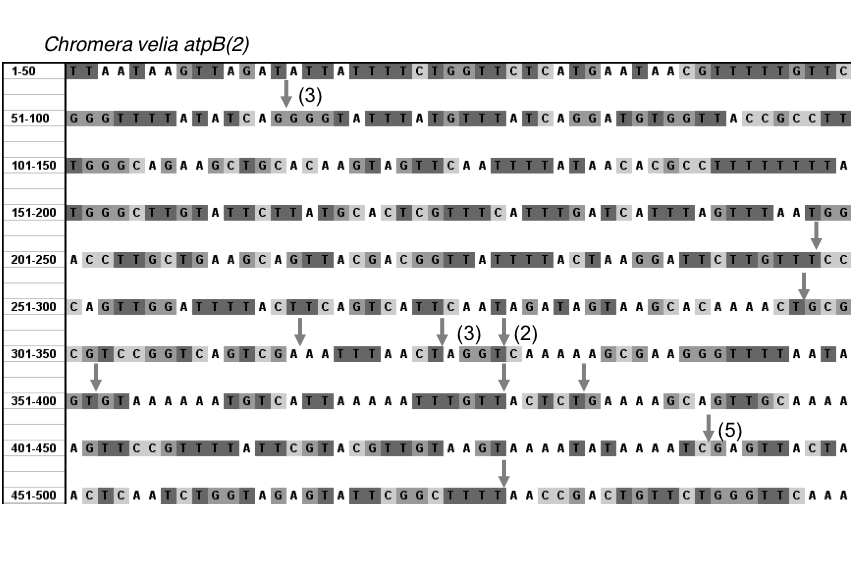

Supplement: Figure S1 — Associated poly(U) sites of the Chromera velia atpB-2 gene. This alignment shows the first 500 bp downstream of the Chromera velia atpB-2 gene. Grey arrows correspond to the different poly(U) sites, identified from the sequences of twenty randomly selected separate, individual cloned oligo-d(A) RT-PCR products using a gene-specific forward PCR primer against C. velia atpB-2. Numbers indicate that multiple colonies gave rise to the same poly(U) site. (TIFF) [file pgen.1004008.s001.tiff]

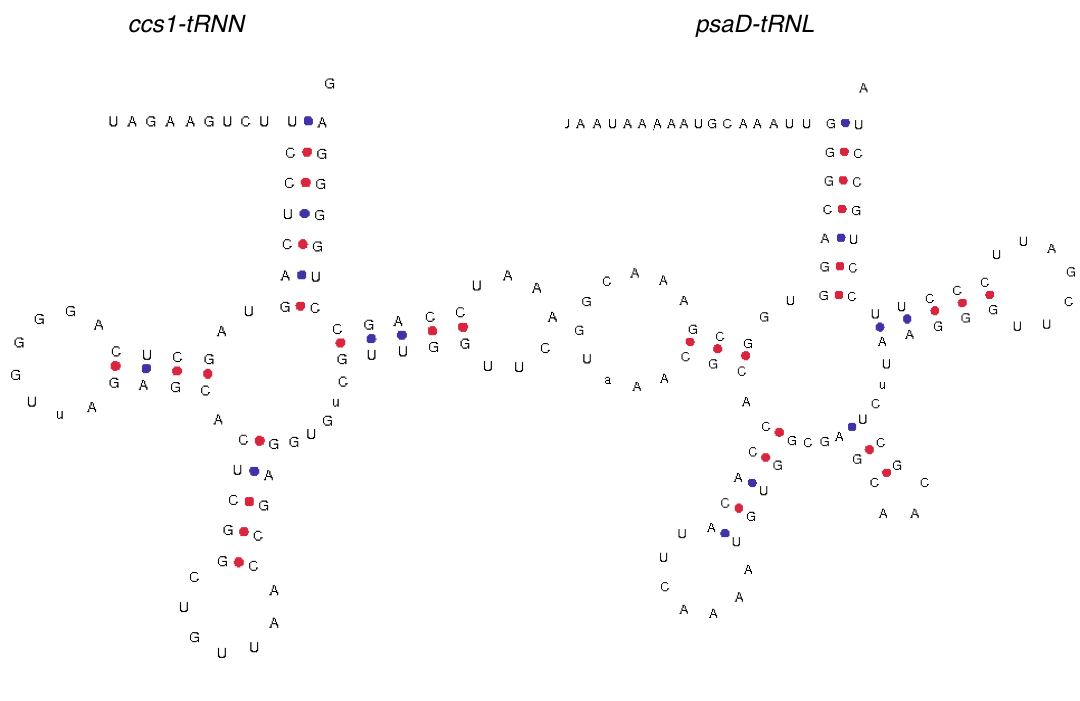

Supplement: Figure S2 — tRNA-associated poly(U) sites in Vitrella brassicaformis. These diagrams show the 3′ UTRs of transcripts for the V. brassicaformis psaD and ccs1 genes, as defined by oligo-d(A) RT-PCR. Grey arrows show the associated poly(U) addition sites for each transcript; the poly(U) tail is not directly shown. In both genes, the poly(U) site is positioned immediately upstream of an associated tRNA (respectively tRNL-CAA and tRNN-GUU). The position and structure of each tRNA, as predicted by the tRNAscan-SE server (http://lowelab.ucsc.edu/tRNAscan-SE/) is shown for each transcript sequence. (TIFF) [file pgen.1004008.s002.tiff]

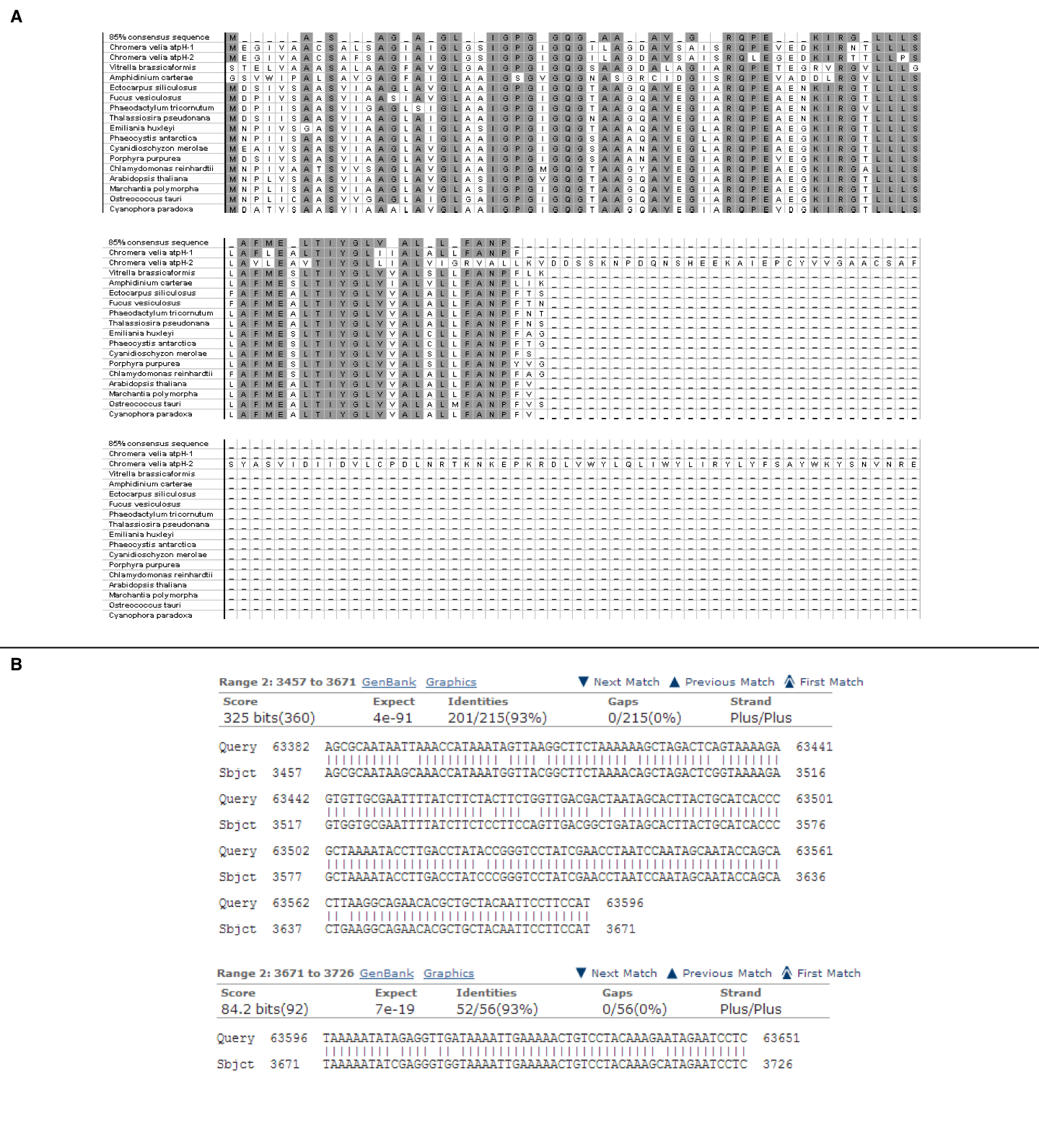

Supplement: Figure S3 — Alignments of chromerid chloroplast atpH sequences. Panel A shows a protein alignment contains the predicted translation products of Chromera velia atpH-1 and atpH-2, and Vitrella brassicaformis atpH, as well as sequences from other representative photosynthetic eukaryotes. Sequence alignments were constructed using MAFFT (http://mafft.cbrc.jp/alignment/server/index.html) using the default settings. An 85% consensus sequence is given at the top of the alignment; characters that match the consensus are shaded for each sequence. The predicted translation product of C. velia atpH-2 contains an 89aa C-terminal extension not found in any other AtpH sequence. Panel B shows nucleotide sequence BLAST alignments for the 5′ end of the coding sequence (i) and 5′ UTR (ii) of the C. velia atpH gene copies. For each alignment, atpH-1 is shown in the query line, and atpH-2 in the subject line. A high degree of sequence conservation (93%) is observed for both regions. This suggests that the very different transcript abundances observed for each gene copy is likely to be dependent on sequence features at the 3′ end of each transcript. (TIFF) [file pgen.1004008.s003.tiff]

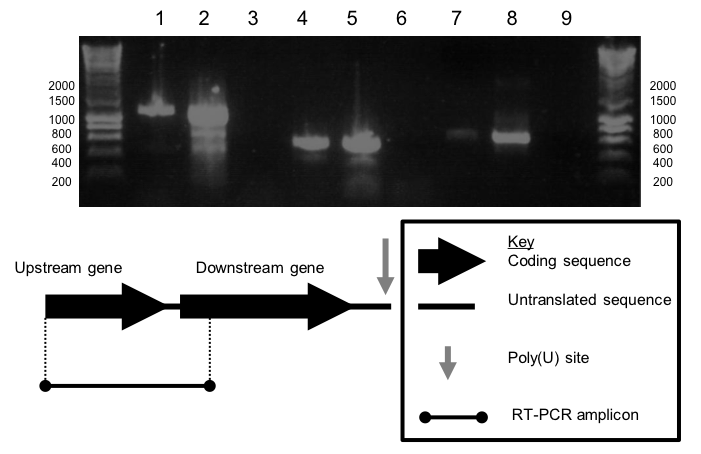

Supplement: Figure S4 — Polycistronic polyuridylylated transcripts in Chromera velia. atpH2-psbA, ORF247-atpB2, and rps14-atpI all consist of an upstream gene that lacks an associated poly(U) site, and a downstream gene that contains an associated poly(U) site as shown in the diagram. Oligo-d(A) cDNA was used as the PCR template, and a PCR was performed to identify dicistronic transcripts, using a forward primer against the 5′ end of the upstream gene, and a reverse primer internal to the downstream gene. PCR over the atpH2-psbA intergenic region using lane 1: oligo-d(A) cDNA; lane 2, gDNA; lane 3, template negative conditions. Lanes 4–6: as lanes 1–3 with ORF247-atpB2 locus. Lanes 7–9: as lanes 1–3 with rps14-atpI locus. (TIFF) [file pgen.1004008.s004.tiff]

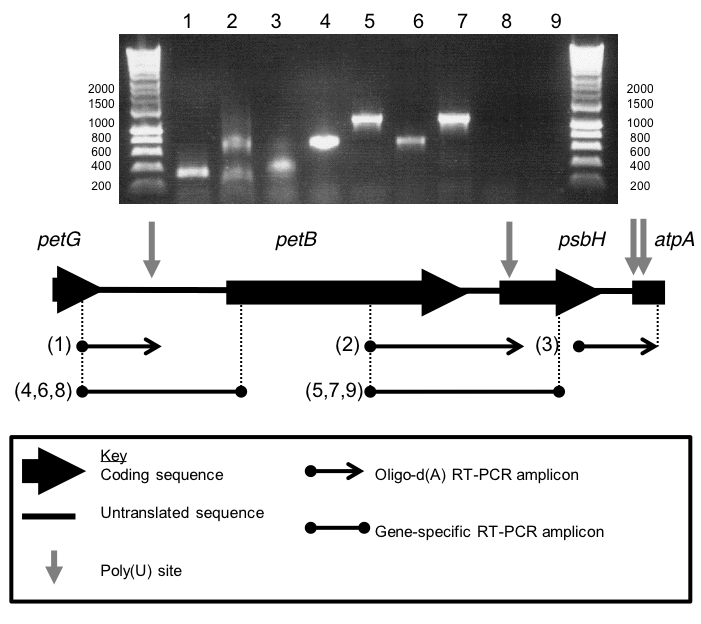

Supplement: Figure S5 — Cotranscription of the Chromera velia petG-petB-psbH locus. As figs. 3 and 4, a transcript diagram with each of the PCR amplicons tested is shown beneath the gel photo. Lanes 1–3: oligo-d(A) RT-PCR for psbH, petB and petG transcripts (all polyuridylylated). The poly(U) sites associated with the petB and psbH genes are positioned respectively inside the 5′ ends of the psbH and atpA coding sequences, hence mature petB, psbH and atpA mRNAs cannot be generated from the same transcript. lanes 4–5: oligo-d(A) RT-PCR for the intergenic petG-petB and petB-psbH regions; lanes 6–7: PCR for the same intergenic regions using DNA template; lanes 8–9: PCR for the same intergenic region using template negative conditions. The positive results for lanes 4–5 indicates that individual poly(U) sites within this locus are generated by alternative 3′ processing of polycistronic precursors. (TIFF) [file pgen.1004008.s005.tiff]
